# Oxidative hypoxia drives TGF-β1–induced fibrosis under normoxia

**DOI:** 10.1016/j.redox.2025.103947

**Published:** 2025-11-26

**Authors:** JinHyuk Choi, Youngmee Kim, Hiruni Nilshi Indeevarie Abeysiriwardhana, Ayusha Malla, Jae-Won Kim, Joshua Miguel Anandappa, Zhuoning Liang, Suyoung Seo, Kwang-Hyeon Liu, Sangkee Rhee, Sang-Soep Nahm, Eui Tae Kim, Yoongho Lim, Moonjae Cho

**Affiliations:** a Department of Biochemistry, College of Medicine, Jeju National University, Jeju, 63241, Republic of Korea; b Jeju Institute of Korean Medicine, Jeju, 63309, Republic of Korea; c Interdisciplinary Graduate Program in Advanced Convergence Technology and Science, Jeju National University, Jeju, 63243, Republic of Korea; d ACHEMBIO Inc., Jeju, 63169, Republic of Korea; e BK21 FOUR Community-Based Intelligent Novel Drug Discovery Education Unit, College of Pharmacy and Research Institute of Pharmaceutical Sciences, Kyungpook National University, Daegu, 41566, Republic of Korea; f Department of Agricultural Biotechnology, Seoul National University, Seoul, 08826, Republic of Korea; g Department of Veterinary Medicine, College of Veterinary Medicine, Konkuk University, Seoul, 05029, Republic of Korea; h Department of Microbiology and Immunology, College of Medicine, Jeju National University, Jeju, 63241, Republic of Korea; i Division of Bioscience and Biotechnology, Konkuk University, Seoul, 05029, Republic of Korea

**Keywords:** Pulmonary fibrosis, Redox signaling, HIF-1α (*HIF1A*), PHD2 (*EGLN1*), NADPH oxidase, Antifibrotic therapy

## Abstract

Pulmonary fibrosis is a progressive and often fatal disease with limited treatment options. Here, we identify a non-hypoxic mechanism for hypoxia-inducible factor 1α (HIF-1α) stabilization as a critical driver of fibrogenesis. Under ambient air conditions (∼18% pericellular O_2_, standard cell culture environment), TGF-β1 activates NADPH oxidase (NOX)2 and upregulates NOX4, generating ROS that oxidize the Fe^2+^ cofactor of prolyl hydroxylase domain-2 (PHD2) and impair HIF-1α hydroxylation. This ROS-mediated pseudo-hypoxic state, which we term “oxidative hypoxia,” promotes a self-reinforcing loop between NOX enzymes and HIF-1α, sustaining fibrosis progression. To counter this process, we developed ACF-2, a rationally designed small molecule that binds PHD2 and scavenges ROS in its microenvironment, thereby preserving PHD2 activity and preventing HIF-1α hyperstabilization. ACF-2 effectively reduced fibrotic markers *in vitro* and attenuated bleomycin-induced pulmonary fibrosis *in vivo*, demonstrating superior efficacy compared with nintedanib. These findings establish oxidative hypoxia as a central mechanism of fibrosis progression and highlight PHD2 as a promising therapeutic target, while introducing ACF-2 as a mechanism-based antifibrotic lead.

## 1 Introduction

Pulmonary fibrosis is a progressive and often fatal lung disease marked by excessive deposition of extracellular matrix (ECM) in the lungs. This leads to alveolar thickening and disruption, impairing gas exchange and often resulting in localized hypoxia [1]. Although antifibrotic agents such as pirfenidone and nintedanib (NIDB) show partial efficacy, therapeutic responses are often limited, and side effects remain a major concern [[2], [3], [4]].

In the hypoxic lungs of patients with pulmonary fibrosis, hypoxia-inducible factor 1α (HIF-1α), the major transcription factor mediating cellular responses to hypoxia [5], is typically upregulated, and its downstream target genes (such as *VEGFA* and *EPO*) are also overexpressed [6] in order to restore oxygen delivery to the fibrotic lung. A similar pattern has been observed in the bleomycin (BLM)-induced animal model, which most closely recapitulates the features of idiopathic pulmonary fibrosis. Traditionally, HIF-1α expression has been regarded as a mere consequence of low-oxygen tension within fibrotic foci [7,8]. However, emerging evidence, including our own, suggests that HIF-1α not only reflects hypoxic conditions but also drives fibrotic progression [9]. Specifically, HIF-1α appears to initiate a positive feedback loop that exacerbates fibrosis even under ambient air conditions (∼18 % pericellular O_2_) [10].

Previous studies have proposed several mechanisms underlying HIF-1α stabilization under ambient air (standard culture) conditions [[11], [12], [13], [14]], focusing primarily on impaired function of prolyl hydroxylase domain-2 (PHD2) [15,16], a key enzyme responsible for hydroxylating HIF-1α and targeting it for ubiquitin-mediated degradation. For instance, nitric oxide has been shown to interfere with PHD2-mediated HIF-1α degradation under normoxia [17], while reactive oxygen species (ROS)-mediated PHD2 dimerization has been reported to reduce its enzymatic activity, leading to HIF-1α stabilization [18]. These studies highlight the critical role of PHD2 in regulating HIF-1α levels, independent of oxygen availability, and underscore the importance of understanding how PHD2 function is modulated in fibrotic conditions.

ROS, especially those generated by NADPH oxidases (NOX) [[19], [20], [21]], are long known to promote fibrotic remodeling. However, their direct molecular targets and the link to HIF-1α stabilization remain unclear. We therefore hypothesized that ROS-driven Fe^2+^ oxidation within PHD2 represents a critical event that compromises PHD2 activity under ambient air conditions, thereby promoting HIF-1α stabilization. To test this, we employed a TGF-β1–induced fibrogenesis model and interrogated whether NOX2 activation and NOX4 upregulation establish a temporal cascade that sustains HIF-1α signaling. This framework guided our biochemical and molecular analyses to delineate a cooperative NOX–ROS–PHD2–HIF-1α axis in fibrotic conditions.

To disrupt this profibrotic network, we adopted a pharmacological strategy aimed at scavenging ROS to restore PHD2 activity and prevent HIF-1α hyperstabilization. To this end, we synthesized a series of candidate compounds incorporating both ROS-scavenging moieties and PHD2-binding capacity. In the present study, we demonstrate that ACF-2, a lead compound from this series, restores PHD2 function and attenuates fibrogenic signaling in cellular and animal models.

Our findings define a previously unrecognized mechanism by which NOX-derived ROS contribute to pulmonary fibrosis through PHD2 inactivation and HIF-1α hyperstabilization. We propose the term “oxidative hypoxia” to describe this state, where oxidative stress functionally mimics hypoxia by disrupting PHD2, leading to aberrant HIF-1α accumulation. This concept provides a mechanistic framework for understanding how ROS-driven signaling sustains fibrosis, independent of oxygen deprivation, and highlights PHD2 as a promising therapeutic target for intervention.

## 2 Results

### 2.1 HIF-1α promotes fibrotic changes

**Fig. 1:**
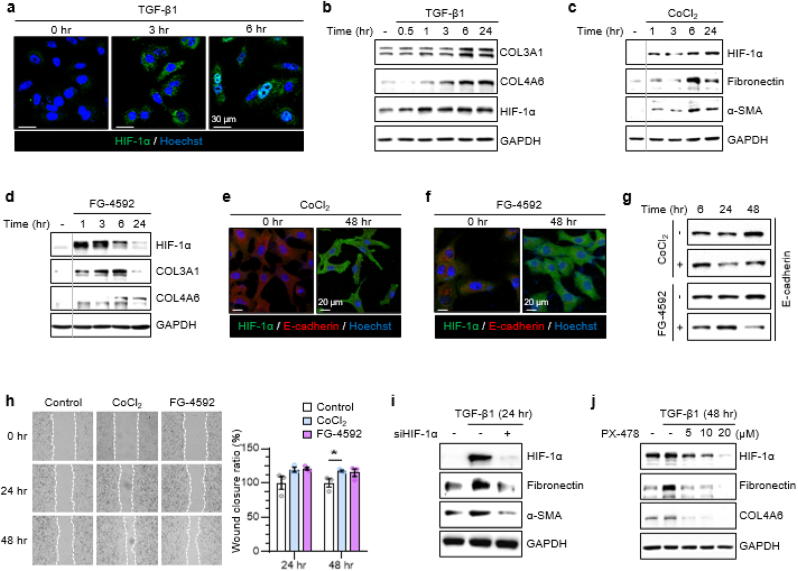
HIF-1α drives fibrotic remodeling in lung-derived cells. (**a**) Immunocytochemistry (ICC) images of A549 show nuclear HIF-1α after 0–6 h TGF-β1 (10 ng/mL). Scale bar, 30 μm. (**b**) Western blot confirms parallel increases in HIF-1α, COL3A1, COL4A6. (**c, d**) Stabilization of HIF-1α using CoCl_2_ (200 μM, **c**) or FG-4592 (50 μM, **d**) increases fibronectin, α-SMA, COL3A1, and COL4A6. (**e, f**) ICC shows that HIF-1α accumulation induced by CoCl_2_ (**e**) or FG-4592 (**f**) is accompanied by reduced E-cadherin, indicative of EMT. Scale bar, 20 μm. (**g**) Western blot validates E-cadherin loss (shared GAPDH with (**c, d**)). (**h**) Migration assay shows greater wound-closure after either HIF-1α stabilizer. (**i**) siHIF-1α (150 nM) blunts TGF-β1-induced fibronectin and α-SMA at 24 h. (**j**) PX-478 co-treatment likewise reduces fibronectin and COL4A6. Data represent mean ± SEM from n = 3 independent cultures performed on different days. For migration assays, horizontal wound distances were quantified using BioTek Cytation 5 software, and group means were compared by one-tailed unpaired Student's t-test in Microsoft Excel (tails = 1, type = 3; ∗p < 0.05). Representative ICC images are from ≥3 independent experiments, each including ≥5 fields per condition.

Multiple studies have reported localized hypoxia and subsequent elevation of HIF-1α levels in the lungs of patients with pulmonary fibrosis [22,23]. Here, we examined the role of HIF-1α in TGF-β1-induced fibrosis using cell lines relevant to lung fibrosis, including alveolar basal epithelial cells (A549), human lung fibroblasts (MRC-5), and bronchial epithelial cells (BBM). Immunocytochemistry showed that intracellular HIF-1α levels increased in response to TGF-β1 stimulation, with nuclear translocation occurring within 6 h (Fig. 1a, see Supplemental Information (SI), Fig. S1a and b). Western blotting further confirmed that HIF-1α upregulation was accompanied by an increase in fibrotic markers, including COL3A1 and COL4A6 (Fig. 1b, see SI, Fig. S1c and d).

Contrary to our initial hypothesis that fibrotic ECM components (e.g., collagen and fibronectin) would accumulate before HIF-1α elevation, Western blot analysis revealed that HIF-1α upregulation precedes ECM deposition. To test whether HIF-1α actively regulates fibrosis under ambient air conditions (∼18% pericellular O_2_), rather than being a downstream consequence of hypoxic ECM accumulation, we stabilized HIF-1α using CoCl_2_ and FG-4592, which inhibit PHD2-mediated hydroxylation and subsequent ubiquitination [24]. Both treatments demonstrated that HIF-1α stabilization led to increased expression of fibrosis-associated proteins, including fibronectin, α-SMA, COL3A1, and COL4A6 (Fig. 1c and d).

To further investigate whether stabilized HIF-1α induces epithelial-mesenchymal transition (EMT), a process closely linked to fibrosis in epithelial cells [25], we examined E-cadherin expression and cell migration following CoCl_2_ or FG-4592 treatment. Immunocytochemistry showed that while HIF-1α levels increased, E-cadherin expression decreased under both treatment conditions (Fig. 1e and f). Western blotting corroborated these findings, confirming the EMT-associated downregulation of E-cadherin (Fig. 1g). In addition, wound healing assays demonstrated a significant increase in cell migration, further supporting EMT induction under CoCl_2_ or FG-4592 treatment (Fig. 1h).

To establish HIF-1α as a key mediator of fibrosis progression, we examined the effects of TGF-β1 treatment following HIF-1α knockdown. In A549 cells transfected with siHIF-1α, the TGF-β1-induced upregulation of fibronectin and α-SMA was markedly attenuated compared to control cells (Fig. 1i). Moreover, pharmacological inhibition of HIF-1α synthesis using PX-478 in TGF-β1-treated A549 cells significantly suppressed fibrosis, as indicated by reduced fibronectin and COL4A6 expression (Fig. 1j, see SI, Fig. S1e and f).

These findings suggest that HIF-1α is not merely a downstream consequence of hypoxia-induced fibrosis but serves as an active driver of early fibrotic progression by promoting fibrosis-specific marker expression and EMT.

### 2.2 PHD2 activity reduction leads to HIF-1α hyperstabilization

**Fig. 2:**
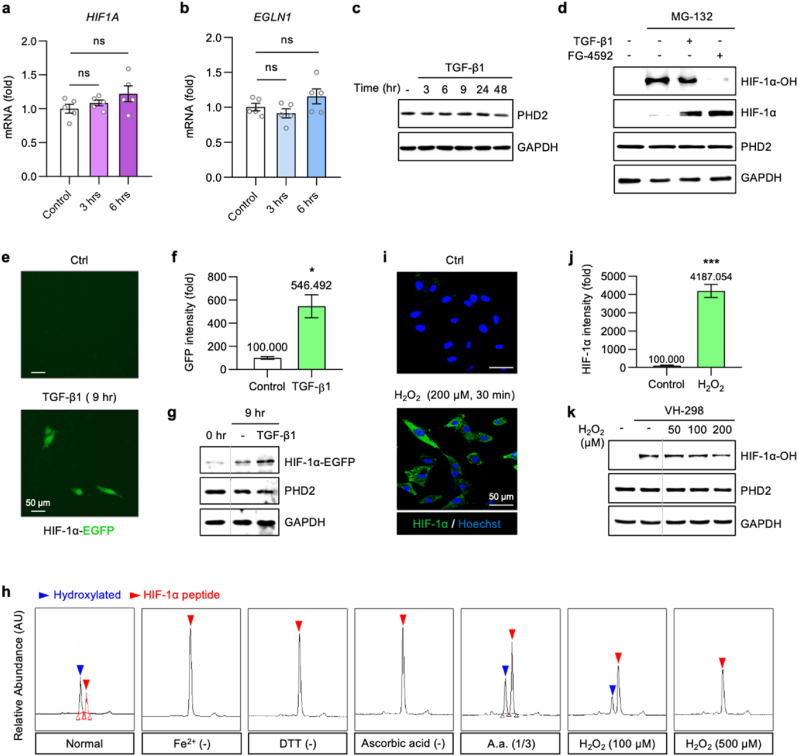
Reduced PHD2 activity leads to TGF-β1–mediated HIF-1α accumulation. (**a**) RT-qPCR shows that HIF-1α mRNA remains unchanged despite protein elevation (see Fig. 1a and b). (**b, c**) PHD2 mRNA and protein levels are largely unaltered, implying post-translational inhibition of PHD2. (**d**) With MG-132 (10 μM), both TGF-β1 (3 h) and FG-4592 (3 h) reduce hydroxylated HIF-1α (HIF-1α-OH), indicating impaired PHD2-mediated hydroxylation. As shown in Fig. 1, TGF-β1 and FG-4592 also increase total HIF-1α levels. **(e, f)** Fluorescence images of pg-HIF-1α-EGFP–transfected A549 show increased signal after TGF-β1 stimulation; quantification is based on identical ROIs applied across all conditions. **(g)** Western blot identifies the ∼27 kDa-larger fusion. (**h**) *In vitro* PHD2 assay shows the hydroxylated HIF-1α peptide peak growing over time but lost without Fe^2+^, DTT, ascorbate, or after H_2_O_2_ addition. (**i, j**) ICC shows nuclear HIF-1α after H_2_O_2_ (200 μM); Scale bar, 50 μm. **(k)** VH-298 assay corroborates oxidation-driven loss of PHD2 activity, leading to reduced HIF-1α-OH following H₂O₂ (1 h) treatment. All experiments were conducted in A549 cells. Data represent mean ± SEM from n = 3 independent biological replicates performed on different days (each with 5 technical replicates for qPCR assays). Fluorescence and ICC intensities were quantified in ImageJ using identical region of interest (ROI)s (≥5 fields per condition) and normalized to the control (100 %). ∗*p* < 0.05, ∗∗*p* < 0.001. Representative fluorescence and ICC images were selected from two independent experiments, each including ≥5 randomly chosen fields per condition.

To determine whether the increased HIF-1α levels that drive fibrosis result from transcriptional upregulation or post-translational stabilization, we first examined HIF-1α mRNA levels in A549, MRC-5, and BBM cell lines following TGF-β1 treatment (Fig. 2a, see SI, Fig. S2a and b). Using real-time qPCR, we analyzed HIF-1α mRNA levels within the time frame corresponding to the previously observed increase in HIF-1α protein levels. While HIF-1α protein levels increased, its mRNA levels did not show a significant change, indicating that HIF-1α accumulation results from impaired degradation rather than *de novo* synthesis.

Next, we sought to clarify the mechanism underlying this stabilization by assessing PHD2 expression, the key enzyme responsible for HIF-1α hydroxylation and subsequent degradation. Neither PHD2 mRNA nor protein levels were significantly altered by TGF-β1 treatment (Fig. 2b and c, see SI, Fig. S2c–f). These findings suggest that the increase in HIF-1α levels is driven by a reduction in PHD2 enzymatic activity rather than a decrease in its expression, leading to HIF-1α hyperstabilization.

To confirm this, we performed two complementary experiments. First, we treated A549 cells with MG-132, a proteasome inhibitor, to block the degradation of ubiquitinated HIF-1α-OH. Western blot analysis revealed that, similar to FG-4592 (a known PHD2 inhibitor), TGF-β1 treatment significantly reduced HIF-1α-OH levels (Fig. 2d). This indicates that TGF-β1 inhibits PHD2-mediated hydroxylation, thereby preventing HIF-1α degradation. Second, we transiently transfected A549 cells with a plasmid encoding HIF-1α-EGFP and monitored GFP intensity under TGF-β1 treatment. Since GFP fluorescence intensity reflects HIF-1α-EGFP stability, its increase indicates reduced PHD2 activity. As expected, TGF-β1 treatment enhanced GFP intensity (Fig. 2e and f), confirming HIF-1α stabilization (Fig. 2g).

Several studies have suggested that TGF-β1 may regulate PHD2 activity indirectly through various signaling pathways [26,27]. To identify whether TGF-β1 directly affects PHD2 enzymatic activity, we performed an *in vitro* PHD2 assay using synthetic HIF-1α peptide as a substrate (Fig. 2h). HPLC analysis confirmed the hydroxylation of synthetic HIF-1α peptide (see SI, Fig. S2g). As the PHD2 reaction progressed, the peak area of the unmodified HIF-1α peptide decreased while the hydroxylated peak (HIF-1α-OH) increased. Importantly, when Fe^2+^ (FeSO_4_), an essential cofactor for PHD2, was omitted, hydroxylation did not occur. Similarly, removing DTT or ascorbic acid, both of which prevent Fe^2+^ oxidation, also inhibited the reaction. These results indicate that the reducing environment provided by DTT and ascorbic acid is crucial for maintaining PHD2 activity by preserving Fe^2+^ in its active state. To further investigate whether Fe^2+^ oxidation impairs PHD2 activity, we introduced H_2_O_2_ into the reaction to promote Fe^2+^ oxidation. H_2_O_2_ inhibited PHD2 activity in a dose-dependent manner, preventing HIF-1α hydroxylation. This effect was confirmed in A549 cells, where H_2_O_2_ treatment increased HIF-1α levels, as demonstrated by immunocytochemistry (Fig. 2i and j) and western blotting (see SI, Fig. S2h). Specifically, treatment with the Von Hippel–Lindau protein antagonist VH-298, which stabilizes hydroxylated HIF-1α by blocking its recognition by pVHL, revealed that stepwise H_2_O_2_ exposure (50–200 μM) led to a dose-dependent reduction in HIF-1α-OH levels, thereby providing direct cellular evidence that PHD2 enzymatic activity is impaired under oxidative stress conditions, even in ambient air (Fig. 2k).

Our findings reveal that TGF-β1 does not reduce PHD2 expression but instead inhibits its enzymatic activity, leading to HIF-1α hyperstabilization and fibrosis progression. This inhibition is linked to Fe^2+^ oxidation and disruption of the cellular redox balance.

### 2.3 NOX-derived ROS contribute to the reduction of PHD2 activity

**Fig. 3:**
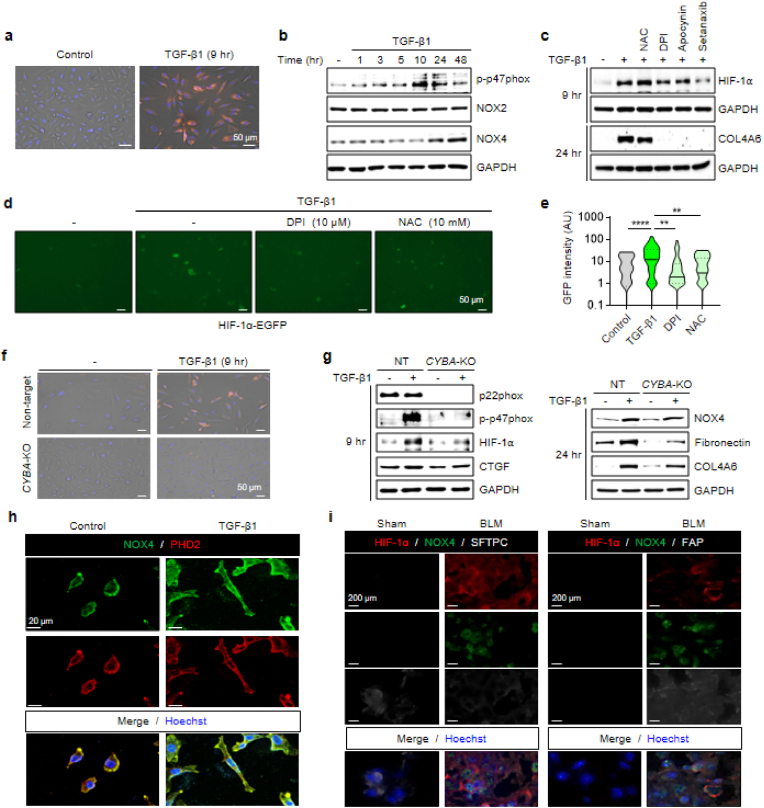
NOX-derived ROS inhibit PHD2 and stabilize HIF-1α (**a**) CellROX™ detects rising ROS accumulation in A549 cells over 9 h TGF-β1; scale bar, 50 μm. (**b**) p47phox phosphorylation and NOX4 up-regulation occur in parallel with HIF-1α elevation (see Fig. 1a and b). Densitometric quantification of p-p47phox, NOX2 and NOX4 normalized to GAPDH is provided in SI, Fig. S3a–c. (**c**) NAC or NOX inhibitors curb HIF-1α and COL4A6, most strongly with DPI, apocynin, setanaxib. Quantified results from three independent experiments are shown in SI, Fig. S3d–e. (**d, e**) Fluorescence images of same cells shown in Fig. 2e–f, treated with TGF-β1 and either DPI or NAC (**d**); scale bar, 50 μm. Both lower fluorescence. The right panel (**e**) quantifies relative GFP intensity from fluorescence images, measured in ImageJ using identical ROIs (≥5 fields per condition) and normalized to the control (100 %). (**f, g**) CRISPR-Cas9 knockout of *CYBA* (encoding p22phox) abolishes TGF-β1–induced ROS (**f**, CellROX™ Deep Red; scale bar, 50 μm), p47phox phosphorylation, HIF-1α, CTGF, NOX4, fibronectin, and COL4A6 (**g**). Quantitative densitometry for the factors is presented in SI, Fig. S3g–m. (**h**) ICC shows that TGF-β1 elevates NOX4 localized adjacent to cytoplasmic PHD2; scale bar, 20 μm. (**i**) Immunofluorescence (IF) staining of lung tissue from BLM-treated (30 mg/kg) ICR mice at 3 weeks post-instillation, compared with sham controls, shows NOX4 and HIF-1α rise in SFTPC^+^ alveolar cells and FAP^+^ fibroblasts; scale bar, 200 μm. Representative IF images were selected from two independent animal experiments, each including ≥5 randomly chosen fields per group. All quantitative data are presented as mean ± SEM from n = 3 independent biological replicates unless otherwise indicated. ∗∗p < 0.01, ∗∗∗∗p < 0.0001.

Previously, we observed that under ambient air conditions, TGF-β1-induced HIF-1α hyperstabilization results from the inhibition of PHD2 activity by ROS, particularly H_2_O_2_. We next sought to determine the mechanism by which TGF-β1 enhances ROS production. It is well established that TGF-β1 promotes intracellular ROS generation, including H_2_O_2_ (Fig. 3a), with multiple potential ROS sources proposed [28,29]. Among these, we focused on NOX isoforms for two primary reasons. First, given that TGF-β1 rapidly induces HIF-1α hyperstabilization, the involvement of a fast-acting ROS source, such as NOX2 [30,31], is highly plausible. Second, stabilized HIF-1α functions as a transcription factor that upregulates its downstream targets, including NOX4 [32], thereby establishing a positive feedback loop in which NOX4-derived ROS further stabilize HIF-1α.

To test these hypotheses, we performed the following experiments. Treatment of A549 cells with TGF-β1, under the same conditions that induced HIF-1α accumulation in Fig. 1a and b, resulted in a rapid increase in p47phox phosphorylation, a key step in NOX2 activation (Fig. 3b). Furthermore, *de novo* synthesis of NOX4 was upregulated at later time points, consistent with transcriptional induction. We next evaluated whether NOX2 and NOX4 contribute to TGF-β1–induced HIF-1α stabilization and fibrosis marker expression by examining the effects of ROS scavengers and NOX inhibitors. Western blot analysis showed that N-acetylcysteine (NAC) and NOX inhibitors (diphenyleneiodonium chloride [DPI], apocynin, and Setanaxib) markedly reduced HIF-1α and COL4A6 levels, with NOX inhibitors displaying a more pronounced effect (Fig. 3c). Similarly, co-treatment of HIF-1α–EGFP-transfected A549 cells with DPI led to a substantial drop in GFP intensity, reinforcing the notion that NOX-derived ROS stabilize HIF-1α (Fig. 3d and e).

To further establish the contribution of NOX-generated ROS to TGF-β1-driven fibrosis, we generated a *CYBA*-knockout (*CYBA*-KO) A549 cell line, in which p22phox, a shared subunit of NOX1–5, was deleted using CRISPR-Cas9. In *CYBA*-KO cells, TGF-β1 failed to generate significant ROS compared with the non-targeting control (Fig. 3f, see SI, Fig. S3f). This is likely because the loss of p22phox impairs p47phox phosphorylation, effectively reducing NOX2-mediated ROS production (Fig. 3g, left panel). As a result, TGF-β1–induced HIF-1α stabilization was markedly diminished (Fig. 3g, left panel), and the upregulation of NOX4 and fibrosis markers (fibronectin, COL4A6) no longer reached the levels seen in non-targeting control cells (Fig. 3g, right panel).

To determine whether membrane-bound NOX4, whose expression is modulated by TGF-β1, could physically interact with cytoplasmic PHD2, we performed immunocytochemical analysis of NOX4 and PHD2 localization following TGF-β1 treatment (Fig. 3h). Z-stack imaging revealed that while PHD2 expression levels remained unchanged, it was predominantly localized to the cytoplasm, excluding the nucleus. In contrast, NOX4 was primarily membrane-bound and exhibited increased expression upon TGF-β1 treatment. Notably, PHD2 was detected in close proximity to NOX4, suggesting that NOX4-derived ROS could directly influence PHD2 function.

Consistent with these findings, NOX4 and HIF-1α upregulation was observed *in vivo* during fibrosis progression (Fig. 3i). Six-week-old ICR male mice were divided into a sham group and a BLM-treated group, with BLM administered via a single intratracheal instillation at 30 mg/kg. Three weeks post-treatment, immunofluorescence analysis revealed a marked increase in NOX4 and HIF-1α levels in the BLM-treated group, particularly within alveolar epithelial type II cells and fibroblasts localized to fibrotic foci, mirroring our *in vitro* observations in A549 cells.

These findings demonstrate that TGF-β1 activates NOX2 and upregulates NOX4 to drive ROS accumulation, leading to PHD2 inhibition and HIF-1α hyperstabilization. This mechanism establishes that HIF-1α can be induced under non-hypoxic conditions through oxidative stress, specifically NOX-derived ROS, thereby extending the concept of chemical hypoxia, where HIF-1α stabilization occurs despite sufficient oxygen availability. By analogy, we define this phenomenon as “oxidative hypoxia”, wherein NOX-derived ROS functionally disrupt PHD2 activity, mimicking hypoxia-induced HIF-1α stabilization without actual oxygen deprivation. Furthermore, fibrosis resulting from this oxidative hypoxia leads to localized tissue hypoxia, further perpetuating HIF-1α stabilization and reinforcing a pathological cycle of pulmonary fibrosis. This self-amplifying fibrotic loop represents a key pathological feature and serves as a potential target for antifibrotic interventions.

### 2.4 ACF-2 enhances PHD2 activity by protecting it from oxidative hypoxia

**Fig. 4:**
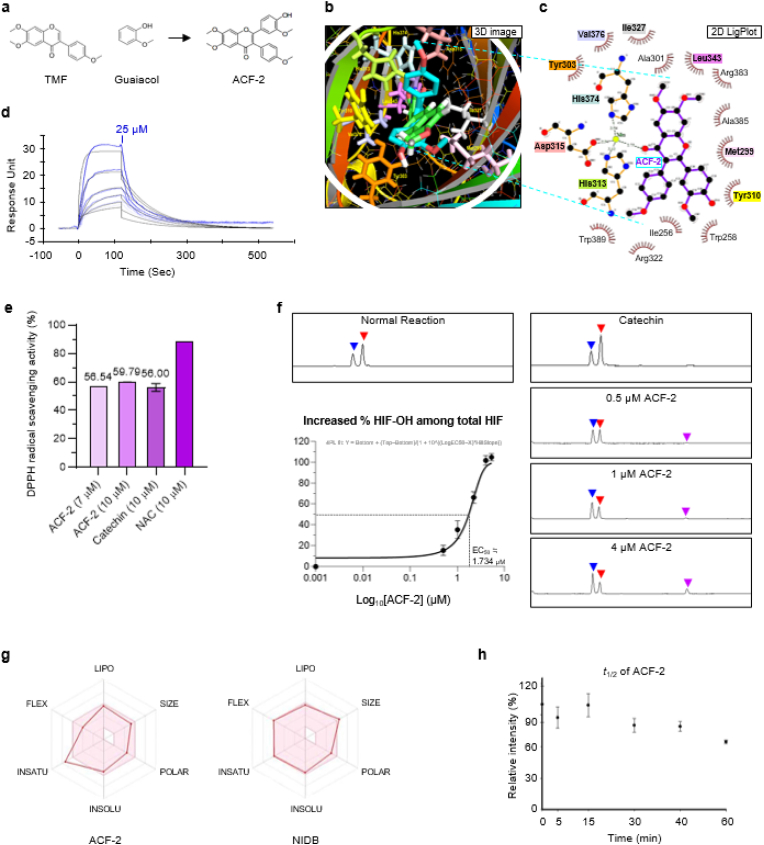
ACF-2 protects PHD2 from oxidative inactivation and exhibits drug-like properties. (**a**) Structure of ACF-2 (TMF core + guaiacol). (**b**) Docking: ACF-2 (cyan) overlaps PW2 (green) in PHD2 active site; shared residues colored. **(c)** LigPlot analysis identifies 13 hydrophobic contacts (red semicircles); Mn-mediated H-bonds replace direct bonds in PW2. (**d**) Surface plasmon resonance (SPR) analysis showing direct binding of ACF-2 (0.78–25 μM) to truncated PHD2. The calculated *K*_D_ is 2.92 μM. (**e**) DPPH assay demonstrates antioxidant activity of ACF-2 compared with NAC and catechin. (**f**) *In vitro* PHD2 enzymatic assay showing that ACF-2 (EC50 approximately 1.734 μM, purple arrow) prevents oxidative inactivation of PHD2. Blue and red arrows indicate the hydroxylated and unmodified HIF-1α peptide peaks, respectively. (**g**) SwissADME profile outperforms nintedanib except for saturation. (**h**) Metabolic stability of ACF-2 in pooled human liver microsomes (HLMs) in the presence of NADPH, and half-life (t_1_/_2_) exceeds 60 min. For the DPPH assay, data represent mean ± SEM from n = 3 independent biological replicates, each including three technical replicates. For the *in vitro* PHD2 assay, data represent mean ± SEM from n = 2 independent biological replicates, each including two technical replicates. For the HLM stability test, data represent mean ± SEM from n = 3 independent biological replicates.

ROS have been extensively studied for their detrimental role in pulmonary fibrosis [[33], [34], [35]]. Given this, NAC, a well-known ROS scavenger, has been evaluated in clinical trials as a potential antifibrotic agent [36,37]. Similarly, previous studies have demonstrated that flavonoid derivatives such as 4′,6,7-trimethoxyisoflavone (TMF) and catechol exhibit antifibrotic properties, primarily attributed to their ROS-scavenging activity [38]. However, rather than focusing solely on ROS neutralization, we aimed to disrupt the fibrotic progression driven by oxidative hypoxia through restoring PHD2 function. To achieve this, we synthesized TMF conjugates with either catechol or guaiacol, the latter being a methoxy-substituted catechol analog that retains ROS-scavenging capacity (Fig. 4a). The objective was to preserve the ROS-scavenging moieties while enabling selective targeting of PHD2. Several candidate compounds were generated by varying the spatial orientation of TMF with catechol or guaiacol, and through *in silico* docking analysis, three potential PHD2-binding candidates (ACF-1, ACF-2, and ACF-3) were identified. Among these candidates, ACF-2 (Fig. 4a, see SI, Fig. S4a) demonstrated the strongest antifibrotic effect, and was therefore selected for further investigation.

To investigate whether ACF-2 could bind PHD2 similarly to known PHD2 inhibitors, we performed an *in silico* docking study using the catalytic domain of PHD2 based on the PDB structure 6yvt. As a positive control, we first re-docked the PHD2 inhibitor spiro[4.5]decanone (“PW2”), reproducing its reported binding interactions and confirming the reliability of our docking procedure (see SI, Fig. S4b and c). We then docked ACF-2—whose 3D conformation was stabilized via molecular dynamics simulations—into the same catalytic domain (Fig. 4b and c). Like PW2, ACF-2 engaged multiple PHD2 residues (Tyr310, Met299, Leu343, Ile327, Val376, Tyr303) in hydrophobic interactions, as well as His313, Asp315, and His374, which in PW2's case form direct hydrogen bonds with PHD2. However, ACF-2 formed no direct hydrogen bonds with PHD2 itself; instead, it interacted through Mn^2+^ and showed a slightly higher binding energy (−6.9 kcal/mol vs. −7.8 kcal/mol for PW2). Despite these differences, the docked model suggests that ACF-2 occupies a similar binding site, supporting our hypothesis that ACF-2 can bind PHD2 and potentially protect it from oxidative inactivation.

Next, we aimed to experimentally validate these computational findings so surface plasmon resonance (SPR) analysis was performed using a truncated form of PHD2 (tPHD2, Pro181–Phe426, 28 kDa) containing its catalytic domain. ACF-2 binding to tPHD2 occurred in a concentration-dependent manner (0.78–25 μM) (Fig. 4d). The *K*_D_ for ACF-2 was determined to be 2.92 μM, significantly lower than ACF-1 (*K*_D_ = 41.7 μM) and ACF-3 (*K*_D_ = 20.3 μM), confirming its superior binding affinity.

Given that oxidative stress impairs PHD2 function, we next examined whether ACF-2 possesses ROS-scavenging properties that could protect PHD2 from oxidative inactivation. A DPPH radical scavenging assay confirmed that ACF-2 retained strong ROS-scavenging activity following its synthetic modification, comparable to catechin and NAC (Fig. 4e). To determine whether this antioxidant effect translated into functional preservation of PHD2, we performed an *in vitro* PHD2 enzymatic assay (Fig. 4f). When 1 μM of PHD2 was used, the half-maximal effective concentration (EC_50_) of ACF-2 was 1.734 μM. In contrast, catechin, a widely recognized antioxidant [39], failed to preserve or enhance PHD2 activity at comparable concentrations, indicating that ACF-2 exerts a distinctive PHD2-protective effect in ambient air. Furthermore, when H_2_O_2_ was introduced to simulate oxidative stress, PHD2 activity was significantly reduced; however, the addition of ACF-2 partially restored its enzymatic function (see SI, Fig. S4d).

In order to evaluate the drug-likeness of ACF-2, we conducted *analysis in silico* ADMET (absorption, distribution, metabolism, excretion, and toxicity) using SwissADME. The physicochemical profile of ACF-2 was assessed across six key parameters━lipophilicity (LIPO), molecular size (SIZE), polarity (POLAR), solubility (INSOLU), saturation (INSATU), and flexibility (FLEX) (Fig. 4g). Compared to NIDB, a clinically approved antifibrotic drug, ACF-2 demonstrated superior performance in multiple categories, although it was slightly below the optimal range in terms of saturation.

To further evaluate the pharmacokinetic properties of ACF-2, we examined its metabolic stability and potential for drug–drug interactions in human liver microsomes (HLMs). When incubated with HLMs in the presence of NADPH, ACF-2 displayed a half-life (t_1_/_2_) exceeding 60 min, indicating that oxidative degradation was negligible and suggesting that ACF-2 is metabolically stable under these conditions (Fig. 4h).

Because inhibition of cytochrome P450 (CYP) enzymes is a major determinant of drug interactions, we next assessed the effect of ACF-2 on nine clinically relevant P450 isoforms (1A2, 2A6, 2B6, 2C8, 2C9, 2C19, 2D6, 2E1, and 3A) using pooled HLMs *in vitro* (Table 1). At concentrations up to 50 μM, ACF-2 showed moderate inhibitory effects against CYP2C8 (IC_50_ = 6.24 μM) and CYP2C19 (IC_50_ = 8.05 μM). In contrast, its inhibitory potential for the remaining seven isoforms was negligible or weak (IC_50_ > 18 μM). These findings suggest that ACF-2 is unlikely to cause clinically significant interactions with the tested CYP isoforms, further supporting its suitability as a therapeutic candidate.

**Table 1:** *In vitro inhibition of nine clinically relevant cytochrome P450 isoforms by ACF-2.* ACF-2 is tested at concentrations up to 50 μM. It shows moderate inhibition of CYP2C8 and CYP2C19, with IC_50_ values of 6.24 and 8.05 μM, respectively. Its effect on other isoforms are negligible or weak, with IC_50_ values greater than 18 μM.

P450 enzymes	Enzyme activity	IC_50_ (μM)
CYP1A2	Phenacetin *O*-deethylation	>50
CYP2A6	Coumarin 7-hydroxylation	>50
CYP2B6	Bupropion hydroxylation	>50
CYP2C8	Amodiaquine *N*-deethylation	8.05 ± 2.26
CYP2C9	Diclofenac 4-hydroxylation	18.13 ± 4.61
CYP2C19	*S*-Mephenytoin 4-hydroxylation	6.24 ± 0.92
CYP2D6	Dextromethorphan *O*-demethylation	40.22 ± 12.36
CYP2E1	Chlorozoxazone 6-hydroxylation	>50
CYP3A	Midazolam 1′-hydroxylation	>50

ACF-2 is a novel dual-function compound that enhances PHD2 activity by scavenging ROS and preventing oxidative hypoxia. Computational and biochemical analyses confirmed its strong binding to PHD2, and enzymatic assays demonstrated protection against oxidative inactivation. ADMET and pharmacokinetic analyses also suggest that ACF-2 exhibits favorable drug-like properties, making it a promising candidate for therapeutic intervention in pulmonary fibrosis by targeting the oxidative hypoxia-driven fibrotic cycle.

### 2.5 ACF-2 prevents *in vitro* HIF-1α hyperstabilization

**Fig. 5:**
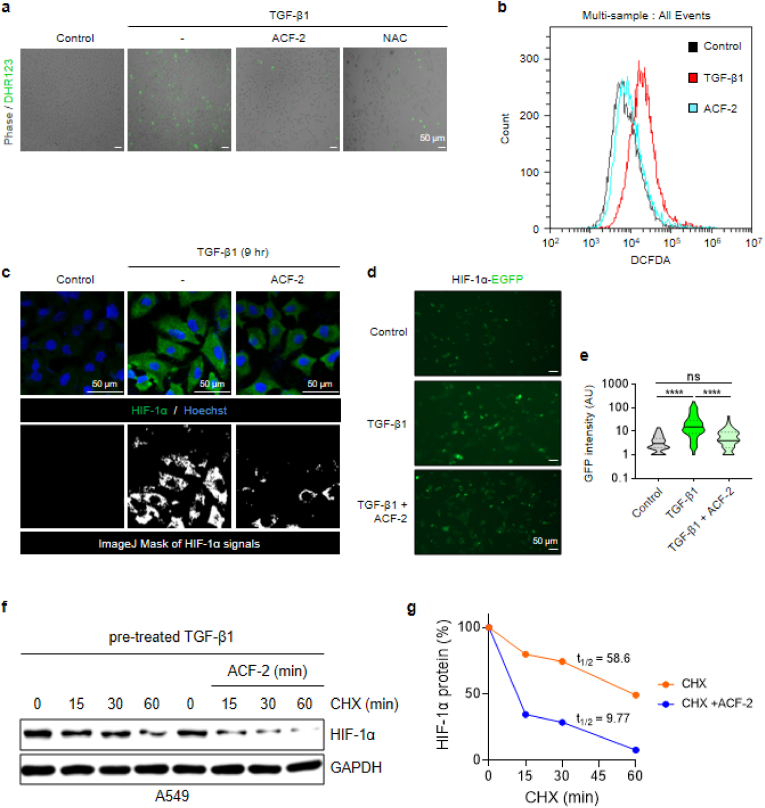
ACF-2 attenuates oxidative hypoxia in cultured cells. (**a**) Intracellular ROS levels visualized by DHR123 staining after TGF-β1 treatment (6 h). ACF-2 reduces ROS to levels comparable to those observed with NAC (10 mM). Scale bar, 50 μm. (**b**) DCFDA-based flow cytometry assay in A549 showing that ACF-2 (7 μM) reduces ROS after TGF-β1 treatment (6 h). (**c**) ICC images of A549 cells show reduced HIF-1α accumulation with ACF-2 co-treatment; scale bar, 50 μm. (**d, e**) Fluorescence imaging of pg-HIF-1α–EGFP–transfected A549 after 9 h TGF-β1 stimulation. Representative images (d) illustrate that ACF-2 diminishes HIF-1α–EGFP signal; scale bar, 50 μm. The right panel (e) shows quantitative analysis of fluorescence intensity measured in ImageJ using identical ROIs (≥5 fields per condition) and normalized to the control (100 %). (**f, g**) Western blot and densitometric analysis of HIF-1α protein stability in A549. (f) Cells were pretreated with TGF-β1 (3 h) and then chased with cycloheximide (CHX, 50 μg/ml) in the presence or absence of ACF-2 (7 μM). (g) Quantification of HIF-1α decay shows that its half-life decreases from 58.6 min (CHX) to 9.77 min (CHX + ACF-2). Fluorescence, ICC, and flow-cytometry data are presented as mean ± SEM from n = 3 independent biological replicates performed on different days. ∗∗∗∗*p* < 0.0001. The CHX chase assay (f, g) was performed once with two technical replicates. Representative fluorescence and ICC images were selected from three independent experiments, each including ≥5 randomly chosen fields per condition.

Previous findings established that ACF-2 not only protects PHD2 from oxidative hypoxia but also possesses drug-like properties with favorable stability. Based on these results, we next sought to evaluate whether ACF-2 exerts antifibrotic effects *in vitro* under standard cell culture conditions (21% O_2_). Prior to these experiments, we performed an MTT assay to confirm that ACF-2 does not induce cytotoxicity at the concentrations intended for *in vitro* studies. Across two cell lines, including A549, ACF-2 did not exhibit cytotoxic effects at concentrations up to 7 μM, which was subsequently used in our *in vitro* assays (see SI, Fig. S5a). To assess whether ACF-2 reduces intracellular ROS, we employed two independent assays. First, using a dihydrorhodamine (DHR)123-based ROS detection assay, we observed that ACF-2 reduced TGF-β1-induced ROS accumulation even more effectively than NAC (Fig. 5a). Second, in a DCFDA-based ROS assay, flow cytometric analysis confirmed that ACF-2 effectively suppressed TGF-β1-induced ROS elevation (Fig. 5b).

We next investigated whether the ROS-scavenging activity of ACF-2 mitigates HIF-1α hyperstabilization. In A549 cells, TGF-β1 treatment markedly increased HIF-1α levels, whereas co-treatment with ACF-2 reduced this effect, as shown by immunocytochemistry (Fig. 5c). A similar trend was observed in A549 cells transfected with a plasmid encoding HIF-1α–EGFP, where ACF-2 significantly attenuated the TGF-β1–induced increase in GFP fluorescence intensity, indicating suppression of HIF-1α stabilization (Fig. 5d and e). To determine whether this effect reflects preserved PHD2 activity, we performed a cycloheximide (CHX) chase assay. Because CHX halts *de novo* synthesis of both HIF-1α and PHD2, any decline in stabilized HIF-1α must reflect degradation by existing PHD2. Under these conditions, HIF-1α decayed much more rapidly in the presence of ACF-2 (t_1_⁄_2_ = 9.77 min) than in its absence (t_1_⁄_2_ = 58.6 min) (Fig. 5f and g), supporting the conclusion that ACF-2 counteracts HIF-1α stabilization by preserving PHD2 activity. Consistently, Western blots showed a dose-dependent decrease in HIF-1α and fibrotic markers in MRC-5 treated with ACF-2 (see SI, Fig. S5b). In addition, we observed similar regulation of HIF-2α: TGF-β1 increased HIF-2α levels in A549 cells, whereas ACF-2 treatment reduced this effect, suggesting that PHD2 protection by ACF-2 may extend to other HIF isoforms that undergo PHD2-dependent hydroxylation in ambient air (see SI, Fig. S5c).

### 2.6 ACF-2 interrupts NOX–HIF-1α feedback loop to suppress fibrosis

**Fig. 6:**
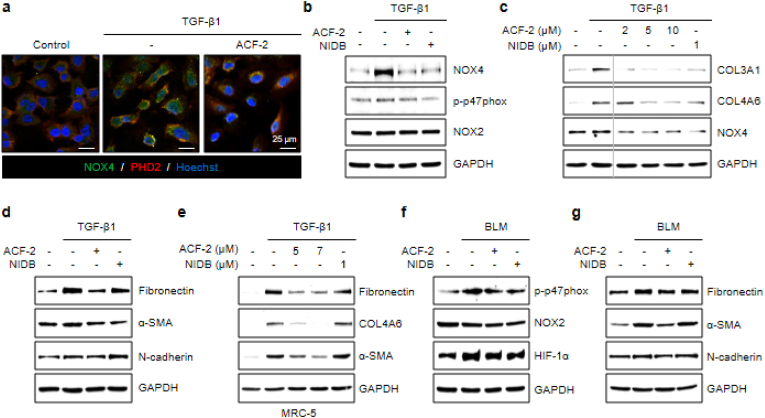
ACF-2 inhibits NOX-derived ROS and HIF-1α signaling to suppress fibrosis. **(a)** ICC showing that ACF-2 curtails NOX4 localization near PHD2 at 24 h TGF-β1. (**b**) Western blot showing that ACF-2 (24 h) diminishes NOX4 and p-p47phox. The effect on NOX2 is relatively minor. (**c**) Western blot showing dose-dependent reduction in COL3A1, COL4A6, and NOX4 under TGF-β1 (24 h). (**d**) At 7 μM, ACF-2 significantly reduces fibronectin, α-SMA, and N-cadherin levels in A549 cells treated with TGF-β1 (24 h). NIDB is included at 1 μM. (**e**) Western blot in MRC-5 cells showing a concentration-dependent decrease in Fibronectin, COL4A6, and α-SMA expression following co-treatment with TGF-β1 and ACF-2. (**f**) Western blot showing that ACF-2 suppresses HIF-1α accumulation and p47phox phosphorylation exposed to BLM (15 μM, 10 h). (**g**) Western blot showing that ACF-2 reduces fibrosis markers, and EMT markers with BLM (24 h). Representative ICC images were selected from two independent biological replicates (each including ≥5 fields per condition). Western blot bands represent one of three independent biological replicates; densitometric quantifications for all Western blots (b–g) are provided in SI, Fig. S6.

Given the predicted specific binding of ACF-2 to PHD2, we hypothesized that ACF-2 also scavenges NOX-generated ROS co-localized with PHD2, which is sufficient to impair PHD2 activity. Consistent with this hypothesis, ACF-2 treatment suppressed the TGF-β1-induced increase in NOX4 expression, which is co-localized with PHD2 (Fig. 6a). Since HIF-1α functions as a transcriptional regulator of NOX4(32), the reduction in NOX4 expression likely results from ACF-2-mediated suppression of HIF-1α levels, a finding further supported by Western blot analysis (Fig. 6b). Notably, while NOX2 expression exhibited a less pronounced reduction, ACF-2 modestly decreased p47phox phosphorylation, suggesting that oxidative stress-induced HIF-1α upregulation reinforces oxidative stress in a positive feedback loop, and that ACF-2 effectively disrupts this pathological cycle.

We proceeded to investigate whether the ACF-2–mediated reduction in ROS and HIF-1α translates into antifibrotic effects, analyzing fibrosis-specific markers by western blotting. In A549 cells treated with TGF-β1, co-treatment with ACF-2 resulted in a dose-dependent reduction of fibrosis markers COL3A1 and COL4A6, which correlated with a similar trend in NOX4 expression (Fig. 6c). Furthermore, at 7 μM, ACF-2 led to a coordinated suppression of fibrosis markers (fibronectin, α-SMA) and EMT marker (N-cadherin), collectively indicating inhibition of fibrosis (Fig. 6d). ACF-2 elicited the same dose-dependent antifibrotic response in MRC-5 cells (Fig. 6e).

To further validate these effects, we induced fibrosis using BLM treatment in A549 cells and examined the antifibrotic efficacy of ACF-2 under these conditions. Similar to TGF-β1 treatment, BLM exposure resulted in a significant increase in HIF-1α levels, which was effectively suppressed by ACF-2 (Fig. 6f). Furthermore, ACF-2 showed a consistent trend toward rescuing BLM-induced elevations in fibrosis marker, myofibroblast activation marker, and EMT marker, supporting its antifibrotic activity under BLM-induced fibrotic conditions (Fig. 6g).

### 2.7 ACF-2 alleviates pulmonary fibrosis *in vivo*

**Fig. 7:**
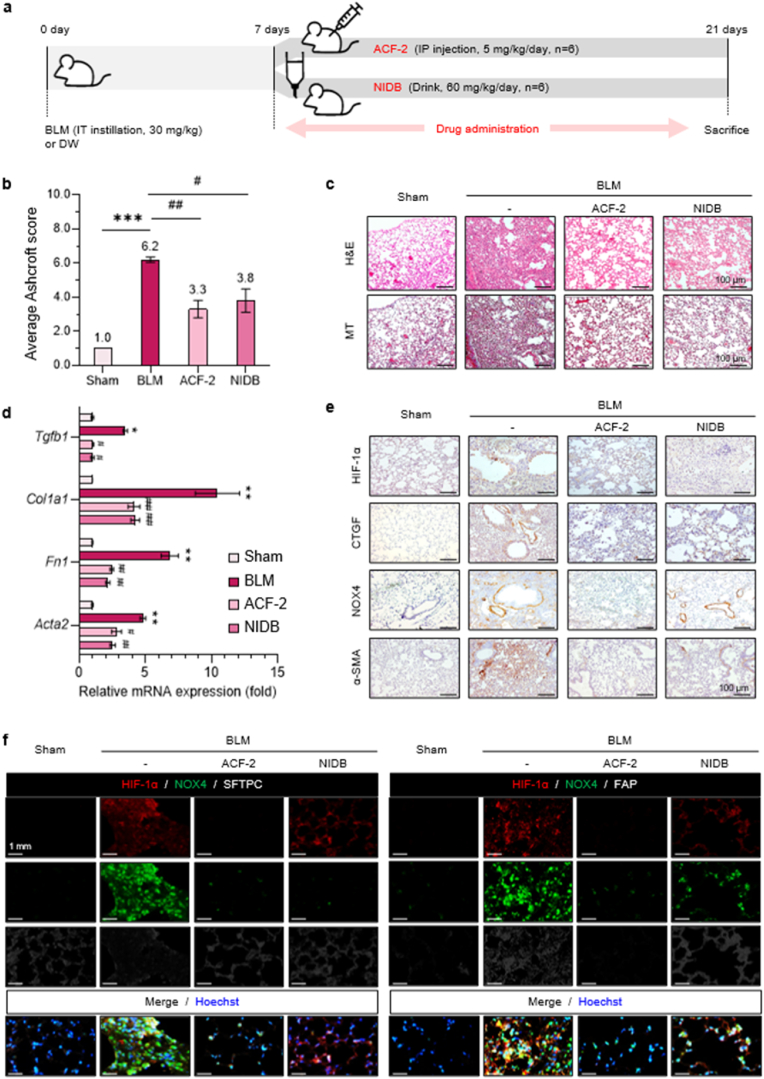
ACF-2 reduces pulmonary fibrosis in BLM-treated mouse model. (**a**) Experimental schematic: intratracheal instillation of BLM (30 mg/kg), followed by treatment with ACF-2 (10 mg/kg every 2 days, intraperitoneal injection in 95 % PBS + 5 % DMSO) or NIDB (60 mg/kg/day, administered in drinking water). Sham and BLM-only groups received corresponding vehicle controls. (**b**) Quantification of fibrosis severity using Ashcroft scores from lung sections of each group (**c**) Representative H&E and MT staining of lung sections show reduced fibrotic changes in ACF-2 and NIDB groups. Scale bars, 100 μm. (**d**) RT-qPCR analysis of lung tissue showing reduced expression of *Tgfb1*, *Col1a1*, *Fn1*, and *Acta2* in treated groups. (**e**) IHC staining of lung sections for HIF-1α, CTGF, NOX4, and α-SMA. ACF-2 and NIDB reduce expression of all four proteins, with ACF-2 showing more pronounced effects on HIF-1α and NOX4. Scale bars, 100 μm. (**f**) Immunofluorescence analysis of lung tissue showing HIF-1α and NOX4 localization in alveolar type II cells (SFTPC positive) and fibroblast (FAP positive). ACF-2 treatment reduces expression of both proteins in both cell types. Representative histological and immunostaining images were selected from six mice per group based on comparable ROI to ensure consistency. Ashcroft score data were obtained from six independent mice per group, and RT-qPCR values represent mean ± SEM from three independent mice per group, each measured in duplicate technical replicates. ∗*p* < 0.05, ∗∗*p* < 0.01, ∗∗∗*p* < 0.001 vs sham; #*p* < 0.05, ##*p* < 0.01, ###*p* < 0.001 vs BLM-only.

Having established its antifibrotic efficacy *in vitro*, we next investigated whether ACF-2 exerts similar therapeutic effects *in vivo*. As outlined in Fig. 7a, a murine model of BLM-induced pulmonary fibrosis was employed to evaluate the effects of ACF-2 treatment. Following sacrifice, the left lobes of the mice were collected and subjected to hematoxylin and eosin (H&E) staining, and the severity of fibrosis was quantified using Ashcroft scoring. The mean fibrosis scores for each experimental group (Fig. 7b) indicate that ACF-2 effectively mitigated BLM-induced fibrosis, exhibiting greater efficacy than NIDB.

Histopathological assessment through H&E and Masson's trichrome (MT) staining (Fig. 7c) further demonstrated that both ACF-2 and NIDB preserved alveolar structures and reduced fibrotic ECM deposition following BLM treatment. These pathological observations were consistent with biochemical analysis━RT-qPCR (Fig. 7d). It revealed that TGF-β1 expression, which was elevated by BLM exposure, was markedly reduced following ACF-2 or NIDB treatment, restoring TGF-β1 levels to near-baseline. Additionally, ACF-2 effectively reversed BLM-induced upregulation of fibrosis markers and myofibroblast activation marker, closely mirroring its *in vitro* effects.

Given the functional interplay between NOX4, HIF-1α, and α-SMA, as well as the involvement of connective tissue growth factor (CTGF), a direct HIF-1α target gene, we further investigated these relationships via immunohistochemistry (Fig. 7e). As anticipated, ACF-2 treatment successfully reduced their elevated expression, supporting its ability to interrupt oxidative hypoxia-driven fibrosis.

Lastly, similar to our previous *in vivo* localization analysis (Fig. 3h), we examined the spatial distribution of HIF-1α and NOX4 in relation to surfactant protein C (SFTPC) and fibroblast activation protein (FAP) in the lungs of BLM-treated mice (Fig. 7f). ACF-2 treatment significantly reduced HIF-1α and NOX4 expression in both alveolar type II epithelial cells and fibroblasts, further reinforcing its mechanistic role in fibrosis suppression.

In addition, assessment of colonic histopathology (see SI, Fig. S7a) showed that, consistent with previous reports of its gastrointestinal adverse effects [40,41], NIDB significantly increased the composite histological score despite reducing lung fibrosis. By contrast, ACF-2 did not induce colonic inflammation or erosion, further underscoring its relative therapeutic advantage.

Collectively, our data demonstrate that ACF-2 alleviates BLM-induced pulmonary fibrosis *in vivo*, consistent with its previously observed mechanism of action and antifibrotic efficacy *in vitro*.

## 3 Discussion

This study identifies oxidative stress as a critical driver of HIF-1α hyperstabilization in pulmonary fibrosis, establishing a mechanistic link between TGF-β1 signaling, NOX-derived ROS, and the functional impairment of PHD2. The concept of “oxidative hypoxia” introduced here describes how oxidative stress disrupts PHD2-mediated HIF-1α degradation, leading to sustained fibrotic signaling even under ambient air conditions. These findings suggest that HIF-1α elevation in fibrosis cannot be attributed solely to local hypoxia; rather, HIF-1α is actively stabilized by ROS, offering a new mechanistic framework for understanding pulmonary fibrosis progression.

In addition to defining this mechanism, our study introduces ACF-2, a mechanism-guided, rationally designed compound that restores PHD2 activity and suppresses oxidative hypoxia. By shielding PHD2 from ROS-induced Fe^2+^ oxidation, ACF-2 prevents HIF-1α hyperstabilization and interrupts the TGF-β–NOX-driven profibrotic loop. By combining mechanistic insights with pharmacological validation, our study emphasizes that PHD2 dysfunction lies at the core of fibrosis and establishes oxidative hypoxia as both a key driver of disease and a promising therapeutic target.

Previous studies reported that TGF-β signaling induces HIF-1α expression through SMAD-dependent mechanisms [8] and that NOX4-derived ROS mediate TGF-β1–induced fibroblast differentiation [21]. Our findings extend these observations by revealing a direct molecular link between NOX-generated ROS and HIF-1α stabilization via PHD2 inactivation, integrating redox regulation into the TGF-β–HIF signaling axis.

Hypoxia has long been implicated in the pathogenesis of fibrotic diseases. The excessive accumulation of fibroblasts, myofibroblasts, and extracellular matrix creates localized hypoxic regions within the lung, making hypoxia a hallmark of idiopathic pulmonary fibrosis (IPF) [42]. In contrast, our data show that HIF-1α acts as an independent driver of fibrotic progression. HIF-1α levels rise before extracellular matrix accumulation, and its stabilization—whether by chemical activation (CoCl_2_, FG-4592) or by ROS-mediated PHD2 inhibition—promotes fibrosis marker expression, whereas its genetic or pharmacological suppression mitigates TGF-β1–induced changes. These findings, consistent with reports that fibroblast-specific HIF-1α deletion attenuates BLM-induced fibrosis [7], highlight HIF-1α stabilization as a pivotal event extending beyond oxygen limitation.

Many studies on fibrotic mechanisms have relied on fibroblasts and focused on their transition into myofibroblasts. In our work, we also used human lung fibroblasts (MRC-5) and bronchial epithelial cells (BBM), but our primary model was the alveolar epithelial type II (AEC II) cell line A549. We chose this cell type for several reasons. First, AEC II, including A549 cells, express both HIF-1α and HIF-2α, making them suitable for studying TGF-β1-HIF-α signaling. Second, pulmonary fibrosis is thought to originate from injury and defective regeneration of AEC II cells [43], meaning that these cells represent a disease-initiating population rather than a downstream responder like fibroblasts. Third, A549 cells allow the study of epithelial–mesenchymal crosstalk, including EMT, which is highly relevant because many HIF-1α target genes are linked to EMT [44]. Finally, A549 cells display relatively robust NOX-related ROS generation, which makes them particularly appropriate for redox biology studies [45]. This feature is especially important for demonstrating the concept of oxidative hypoxia, where epithelial cells provide a more direct model than fibroblasts.

In addition to HIF-1α, previous studies have also implicated HIF-2α in the pathogenesis of pulmonary fibrosis [42,46,47]. HIF-2α has been associated with chronic hypoxia–driven responses, including sustained metabolic reprogramming, vascular remodeling, maladaptive epithelial repair, and fibroblast activation, whereas genetic or pharmacologic inhibition of HIF-2α attenuates fibrosis [46]. Our study focused primarily on HIF-1α, which we propose functions as an early mediator in the initiation of fibrotic remodeling. Unlike HIF-2α, which appears to act in the later and chronic phase of disease, HIF-1α is rapidly stabilized in response to acute oxidative and hypoxic stress and triggers profibrotic pathways such as TGF-β1–induced epithelial–mesenchymal transition. Moreover, HIF-1α is broadly expressed across diverse pulmonary cell types, including alveolar epithelial cells, fibroblasts, and bronchial epithelial cells, whereas HIF-2α expression is more restricted to specific populations such as type II alveolar epithelial cells [[48], [49], [50]]. Consistent with this broader distribution, we observed that ROS-mediated HIF-1α stabilization occurred in all three cell types used in our study. Together, these considerations highlight the importance of HIF-1α as an initiating driver of fibrosis while acknowledging a limitation of our work: the precise contribution of HIF-2α remains to be defined. Future studies dissecting the relative roles of HIF-1α and HIF-2α will be critical to fully understand how hypoxia signaling orchestrates fibrotic progression.

While previous studies have reported that TGF-β1 signaling can suppress PHD2 expression at both the mRNA and protein levels [8,27], we did not observe significant changes in PHD2 expression following TGF-β1 treatment in A549 cells. Notably, McMahon et al. [27] described a mechanism in which TGF-β1–induced pSMAD2/3 directly downregulated PHD2 expression, thereby stabilizing HIF-1α. In contrast, our findings indicate a post-translational mechanism in which oxidative stress impairs PHD2 enzymatic activity through Fe^2+^ oxidation. This observation is consistent with the established effects of metal ion oxidation on the activity of metalloenzymes such as superoxide dismutase 1 and catalase [51,52]. In addition, previous studies have shown that oxidation of critical cysteine residues in PHD2 promotes its dimerization and reduces enzymatic activity, thereby contributing to HIF-1α accumulation [18].

Our study extends these findings by demonstrating that Fe^2+^ oxidation provides a rapid and direct route to PHD2 inactivation, linking a simple chemical event to the stabilization of HIF-1α. Acting independently of transcriptional or translational regulation, this mechanism offers a clear explanation for how oxidative stress drives fibrosis. Collectively, our data identify PHD2 as a key enzymatic target of ROS and define its oxidative inactivation as a principal mechanism sustaining HIF-1α signaling and fibrosis.

ROS have long been implicated in fibrosis, with numerous studies reporting that oxidative stress arising from environmental toxins, mitochondrial dysfunction, or NOX4 activation promotes epithelial injury, fibroblast activation, and extracellular matrix deposition [34,53]. For example, Kamp et al. [54] demonstrated that ROS directly damage alveolar epithelium and drive fibroblast activation, whereas Hecker et al. [53] highlighted their role in enhancing TGF-β1 activation and NOX4-dependent profibrotic signaling, thereby promoting EMT and fibroblast-to-myofibroblast differentiation. In this context, we identify NOX-derived ROS as the primary mediators of oxidative hypoxia in fibrotic conditions. While mitochondrial ROS have also been implicated in TGF-β1 signaling [55,56], accumulating evidence highlights NOX4-generated ROS as the predominant driver of fibrosis [21]. This is attributable to several unique features of NOX4: it is directly induced by TGF-β1, produces sustained H_2_O_2_ output rather than transient bursts [57], and is consistently required for myofibroblast differentiation and extracellular matrix deposition in experimental models. Our previous work also demonstrated that TGF-β1 upregulates NOX4 expression, and that NOX4-derived ROS are critical for EMT progression, extracellular matrix remodeling, and cell proliferation [58].

Consistent with these properties, our findings demonstrate that NOX enzymes provide a rapid and direct source of ROS in fibrogenesis. Specifically, NOX2 was rapidly activated through p47phox phosphorylation, whereas NOX4 was upregulated at later stages, establishing a positive feedback loop that further stabilized HIF-1α. This interplay between NOX-generated ROS and HIF-1α created a self-sustaining cycle of fibrosis, which we validated using NOX inhibitors and CRISPR-mediated *CYBA*-KO. Moreover, *in vivo* analyses revealed that NOX4 and HIF-1α were both upregulated in BLM-induced fibrosis, particularly in alveolar epithelial cells and fibroblasts. Importantly, the co-localization of NOX4 and PHD2 suggests that NOX-derived ROS directly impair PHD2 function, thereby reinforcing oxidative hypoxia as a central driver of fibrogenesis.

These findings underscore the need for therapeutic strategies that can restore PHD2 activity without globally suppressing ROS. To this end, ACF-2 was developed. It should be noted that ACF-2 is not selective for a particular ROS species or source; rather, its specificity arises from its biochemical target, PHD2. By directly protecting PHD2 from ROS-driven Fe^2+^ oxidation, ACF-2 preserves enzymatic activity and interrupts the downstream profibrotic signaling cascade without indiscriminate ROS scavenging. This strategy contrasts with general antioxidants such as NAC, which require high concentrations and indiscriminately reduce both physiological and pathological ROS, thereby increasing the risk of off-target effects. In comparison, ACF-2 is designed to preserve normal redox signaling while selectively preventing PHD2 inactivation. Its targeted scavenging activity enables effective antifibrotic action at much lower concentrations (7 μM vs. the ∼10 mM typically required for NAC *in vitro*). Importantly, by avoiding global ROS depletion—some of which are essential for normal signaling [59,60]—ACF-2 specifically neutralizes the pathological ROS that disrupt PHD2. This selectivity not only distinguishes it from nonspecific scavengers such as NAC and NOX inhibitors like setanaxib, but also from existing antifibrotic agents. Unlike NIDB, which broadly inhibits receptor tyrosine kinases and interferes with normal vascular and epithelial repair [61], ACF-2 targets a more defined molecular pathway, potentially reducing systemic toxicity. This distinction was further supported by our *in vivo* colon histopathology, where NIDB significantly increased inflammation and erosion scores, whereas ACF-2 alleviated pulmonary fibrosis without inducing detectable gastrointestinal side effects.

Beyond its effects on HIF-1α, it will be important to examine whether ACF-2 also influences HIF-2α, which has been implicated in chronic hypoxia–driven profibrotic signaling and maladaptive epithelial repair. Our *in vitro* experiments showed that ACF-2 partially reversed TGF-β1–induced upregulation of HIF-2α, likely through its ability to restore PHD2 activity. Since both PHD2 and PHD3 contribute to HIF-2α hydroxylation [62], future studies should clarify whether ACF-2 also modulates PHD3, thereby extending its therapeutic relevance beyond the initiation phase of fibrosis. In line with these considerations, the translational potential of ACF-2 requires further validation, particularly with regard to its long-term pharmacokinetics, bioavailability, and possible off-target effects in rigorous preclinical models.

Collectively, these findings establish a mechanistic rationale for targeting PHD2 as an antifibrotic strategy and position ACF-2 as a promising lead compound for further development. Importantly, ACF-2 functions as an agonist that restores PHD2 activity to physiological levels, restraining excessive HIF-1α accumulation while preserving normal regulation. Evaluating ACF-2 in combination with current antifibrotic therapies—such as NIDB or pirfenidone—may further clarify whether it provides additive or synergistic benefits. By disrupting the pathological cycle of oxidative hypoxia while maintaining physiological HIF-1α regulation, ACF-2 has the potential to serve as the basis for future therapeutic approaches aimed at halting or even reversing pulmonary fibrosis.

## 4 Materials and methods

Full experimental protocols, primer and Guide-RNA (gRNA) sequences, and complete vendor information (manufacturer + catalog no.) are compiled in the **Appendix: Supplemental Information** file, section “**② Document S2. Protocols & Materials (**Tables S1–S26**)**”. Below, only the essential conditions required for interpretation of the data are retained.

### 4.1 Cell lines and culture

A549, MRC-5, and BBM cells (ATCC) were grown in DMEM (4.5 g/L d-glucose) or MEM (1 g/L d-glucose) with 10 % FBS and 1 % penicillin-streptomycin at 37 °C in a humidified incubator with 5 % CO_2_ and ambient air (∼18 % pericellular O_2_). Serum was lowered to 2 % for routine assays and to 0.1 % for siRNA transfection.

### 4.2 Combination treatment

Cells were exposed to TGF-β1, BLM, CoCl_2_, FG-4592, ACF-2, and pathway modulators at the doses and time points indicated in the figure legends; vehicle concentrations were matched.

### 4.3 RNA Interference

Cells were transfected with 5 nM siHIF-1α or non-targeting siRNA (INTERFERin®) and harvested 48 h later.

### 4.4 Transient transfection (pg-HIF-1α–EGFP)

pg-HIF-1α–EGFP was introduced via jetOPTIMUS®; GFP fluorescence was quantified 48 h post-transfection.

### 4.5 CRISPR/Cas9-mediated CYBA knockout in A549 cells

Two *CYBA*-targeting gRNAs were cloned into PX459. A549 cells were puromycin-selected, single-cell cloned, and screened for p22phox loss by Western blot [63].

### 4.6 Protein extraction and western blot analysis

RIPA lysates (BCA-normalized) were resolved by SDS-PAGE, transferred to nitrocellulose, and probed with the antibodies listed in Appendix S2**. Protocols & Materials**.

### 4.7 Migration assay

Scratch wounds (AutoScratch™) were imaged at 0, 6, 24 and 48 h on Cytation 5; wound width was quantified automatically.

### 4.8 RNA Isolation and RT-qPCR

Total RNA (AccuPrep®) was reverse-transcribed and amplified with SYBR Green chemistry on a StepOnePlus™ system; relative expression was calculated by ΔΔCt.

### 4.9 MTT assay

Cells (8 × 10^3^ per well, 96-well) were treated as indicated, incubated with 0.5 mg/mL MTT for 4 h, solubilized in DMSO, and read at 570 nm.

### 4.10 ROS staining

CellROX™ Deep Red or DHR123 fluorescence microscopy, and CM-H_2_DCFDA flow cytometry, were used to quantify intracellular ROS.

### 4.11 DPPH assay

Equal volumes of 100 μM DPPH and test compound (7–10 μM) were incubated 30 min (dark); absorbance at 517 nm yielded % scavenging.

### 4.12 Surface plasmon resonance (SPR)

His-tPHD2 was immobilized on an HC1000 chip; ACF-2 (0.78–25 μM) flowed at 50 μL/min. Sensorgrams were fitted to a 1:1 model to obtain *K*_D_.

### 4.13 In vitro PHD2 assay

Recombinant PHD2 (1 μM) hydroxylated HIF peptide (300 μM) ± ACF-2; substrate and product peaks were resolved by HPLC.

### 4.14 In vitro pharmacokinetics

ACF-2 metabolic stability (1 μM, 0–60 min) and CYP inhibition (0–50 μM) were determined in pooled human liver microsomes and quantified by LC-MS/MS [64,65].

### 4.15 In silico 3D binding

ACF-2 was docked to MD-relaxed PHD2 (PDB 6yvt) [66] with AutoDock Vina [67]; interactions were visualized in PyMOL and LigPlot [68,69].

### 4.16 Computational prediction of ADME properties

SwissADME [70] was used to predict key physicochemical and pharmacokinetic parameters (logP, solubility, GI absorption, BBB penetration, P-gp/CYP interactions, etc.) for ACF-2 and nintedanib; full settings and outputs are provided in Appendix S2**. Protocols & Materials.**

### 4.17 Nuclear Magnetic Resonance (NMR)

ACF-2 (∼50 mM, D_2_O or DMSO‑*d*_6_) was analyzed on a 400 MHz spectrometer; chemical shifts were assigned with SPARKY 3 [71].

### 4.18 Mice experiment

Male ICR mice received BLM (30 mg/kg, intratracheal). ACF-2 (10 mg/kg IP, q48 h) or nintedanib (60 mg/kg PO, qd) was given for 2 wk (Jeju National University IACUC approval).

### 4.19 Histological examinations and scoring

Fixed left lung lobes were paraffin-embedded, sectioned (4 μm), and stained with H&E or MT. A blinded pathologist graded pulmonary fibrosis on the 1–8 Ashcroft scale; representative images illustrate typical lesions. Colon samples were processed by the swiss-roll method, stained with H&E, and scored for inflammation and erosion, which were combined into a composite histological score.

### 4.20 Immunohistochemistry

Sections underwent antigen retrieval, HRP detection of primary antibodies (HIF-1α, CTGF, NOX4, α-SMA), and hematoxylin counterstaining.

### 4.21 Immunofluorescence

Cultured cells and tissue sections were probed with primary antibodies, Alexa Fluor® secondaries, and imaged by confocal microscopy.

### 4.22 Statistical analysis

Statistical analyses were performed using Microsoft Excel and GraphPad Prism 8. Data are presented as mean ± SEM. One-tailed unpaired Student's t-tests (Excel T.TEST, type = 3, tails = 1) were used for pairwise comparisons, and one-way ANOVA with Sidak's multiple-comparisons test was applied where appropriate.

## CRediT authorship contribution statement

**JinHyuk Choi:** Data curation, Formal analysis, Investigation, Methodology, Validation, Visualization, Writing – original draft, Writing – review & editing. **Youngmee Kim:** Conceptualization, Investigation. **Hiruni Nilshi Indeevarie Abeysiriwardhana:** Data curation, Formal analysis, Investigation, Validation, Visualization. **Ayusha Malla:** Investigation. **Jae-Won Kim:** Investigation, Resources. **Joshua Miguel Anandappa:** Investigation. **Zhuoning Liang:** Data curation, Formal analysis, Investigation. **Suyoung Seo:** Investigation, Resources. **Kwang-Hyeon Liu:** Methodology, Resources, Writing – review & editing. **Sangkee Rhee:** Methodology, Resources. **Sang-Soep Nahm:** Data curation, Investigation. **Eui Tae Kim:** Formal analysis, Investigation, Resources, Writing – review & editing. **Yoongho Lim:** Conceptualization, Formal analysis, Methodology, Software, Supervision, Validation, Writing – review & editing. **Moonjae Cho:** Conceptualization, Funding acquisition, Methodology, Project administration, Resources, Supervision, Validation, Writing – review & editing.

## Declaration of competing interest

The authors declare no competing interests.

## Data Availability

Data will be made available on request.
