# Supplementary material for: Oxidative hypoxia drives TGF-β1–induced fibrosis under normoxia
Source: Redox Biol. 2025 Nov 26;89:103947. doi: 10.1016/j.redox.2025.103947 (PMC12721191; doi:10.1016/j.redox.2025.103947)
Supplement: Multimedia component 1 [file mmc1.docx]

**Appendix: Supplemental Information**

**① Document S1. Figures S1-S7**

***Figure S1.*** *Additional validation of hypoxia-inducible factor 1α (HIF-1α) elevation in fibrotic lung cell models.*

(**a, b**) ICC of human lung fibroblasts (MRC-5, **a**) and bronchial epithelial cells (BBM, **b**) treated with TGF-β1 (10 ng/mL) for up to 9 hr, illustrating the nuclear accumulation of HIF-1α (green). Nuclei are stained with Hoechst 33342 (blue). Scale bar, 50 µm for (**a**), 20 µm for (**b**). (**c, d**) Western blot analyses in MRC-5 (**c**) and BBM (**d**) confirm that TGF-β1 treatment elevates HIF-1α along with fibrotic markers (COL3A1, fibronectin, α-SMA, and/or COL4A6). (**e, f**) Additional data show that CoCl₂ (**e**) or FG-4592–induced (**f**) HIF-1α stabilization in A549 cells is mitigated by the HIF-1α transcriptional inhibitor PX-478, thereby reducing fibrosis-associated proteins. Representative ICC (including ≥ 5 randomly chosen fields per condition) and Western blot images were selected from 3 independent biological experiments.

***Figure S2.*** *Further evidence of decreased prolyl hydroxylase domain-2 (PHD2) activity under TGF-β1 and oxidative stress*.

(**a, b**) Real-time qPCR (RT-qPCR) of HIF-1α in MRC-5 (**a**) and BBM (**b**) at 0–6 hr post–TGF-β1 (10 ng/mL) treatment shows minimal or no increase in HIF-1α mRNA compared to robust protein induction. (**c–f**) Extended mRNA and Western blot analyses of PHD2 in MRC-5 (**c, e**) and BBM (**d, f**) treated with TGF-β1 (up to 6 hr for mRNA and up to 24 hr for protein), confirm that TGF-β1 has no significant effect on PHD2 expression levels. Bars show the mean±SEM calculated from four independent biological samples in **a-d**. (**g**) *In vitro* PHD2 assay (performed under ambient air culture conditions) validates the identifies of synthetic HIF-1α and HIF-1α-OH peptides. Under our HPLC conditions, the synthetic HIF-1α peptide is detected at 17.128 min, while HIF-1α–OH appears at 16.810 min. This retention pattern is consistently observed across all *in vitro* PHD2 assays conducted in this study. (**h**) Western blot analysis of HIF-1α from cells treated to H₂O₂ (200 μM). Representative Western-blot images were selected from 3 independent biological replicates. RT-qPCR data were obtained from n = 3 independent biological cultures (each measured 4 technical replicates) and analyzed using a one-tailed unpaired Student’s t-test (tails = 1, type = 3; unequal-variance assumption). All values are presented as mean ± SEM. p < 0.05 was considered statistically significant.

***Figure S3.*** *Additional evidence for NADPH oxidase-related ROS, based on validated CYBA-knockout (CYBA-KO) cells.*

(**a–e, g-m**) Densitometric quantification of Western blots presented in Figure 3b,c and g, showing relative expression of the indicated proteins normalized to GAPDH and then to the control (100%). Each bar represents data from three independent biological replicates obtained from separate cultures. (**f**) Fluorescence-based detection of ROS using using DHR123 in *CYBA*-KO cells, compared to non-targeting control cells, shows significantly reduced ROS levels following TGF-β1 treatment. Scale bar, 50 µm. Representative images were chosen from two independent experiments, each including ≥ 5 random fields per condition. All densitometric data (a–e, g–m) were analyzed using a one-tailed unpaired Student’s t-test (tails = 1, type = 3; unequal-variance assumption). All values are expressed as mean ± SEM. **p* < 0.05, ***p* < 0.01.

***Figure S4.*** *Additional data on ACF-2 synthesis, structure, and PHD2 protection.*

(**a**) Representative ¹H-NMR, ¹³C-NMR, and mass spectrometry (MS) spectra of ACF-2. The MS spectrum shows a peak corresponding to the sodium adduct [M + Na]^+^ at m/z ~ 456.6. These data confirm the expected molecular mass and support the structural integrity and purity of ACF-2 (molecular weight: 434.44). (**b, c**) Chemical structure of 2-[[5-(6-methoxynaphthalen-2-yl)-3-oxidanyl-pyridin-2-yl]carbonylamino]ethanoic acid (PW2) (**b**), and LigPlot analysis of the PHD2 catalytic domain bound to PW2 (**c**). Hydrophobic interactions with Tyr310, Met299, Tyr303, Ile327, Leu343, and Val376 are denoted with red semicircles. Hydrogen-bonds with Tyr329, Arg383, His374, Asp315, and His313 are marked with green dotted lines. (**d**) *In vitro* PHD2 assay performed under oxidative conditions with exogenous H₂O₂ (20 µM). Addition of ACF-2 (4 µM) restores enzymatic activity.

***Figure S5.*** *ACF-2 attenuates TGF-β1–induced HIF-α stabilization without cytotoxicity.*

(a) MTT assay showing that ACF-2 (7 µM) does not induce cellular cytotoxicity, with or without TGF-β1. (**b**) Western blot in MRC-5 cells showing a dose-dependent reduction in HIF-1α levels after 9 hr TGF- β1 treatment with ACF-2. (**c**) Western blot in A549 cells showing that ACF-2 reduces HIF-2α accumulation at 12 hr and 15 hr following TGF-β1 stimulation. Representative Western-blot images were selected from three independent biological replicates. MTT assay data were obtained from four independent biological samples and analyzed using a one-tailed unpaired Student’s t-test (tails = 1, type = 3; unequal-variance assumption). All values are expressed as mean ± SEM, and p < 0.05 was considered statistically significant.

***Figure S6.*** *Quantitative analysis of Western-blot data supporting Figure 6*

(**a-r**) Densitometric quantification of Western blots presented in Figure 6 (b–g), showing the relative expression of the indicated proteins normalized to GAPDH and to the control (100%). Panels (a–i) correspond to TGF-β1–treated A549 cells, while panels (j–r) correspond to BLM-treated cells. Each bar represents data from three independent biological replicates, each obtained from separate cultures or animals. All densitometric values were analyzed using a one-tailed unpaired Student’s t-test (tails = 1, type = 3; unequal-variance assumption). Data are presented as mean ± SEM; **p* < 0.05, ***p* < 0.01 and ****p* < 0.001.

***Figure S7.*** *ACF-2 attenuates bleomycin (BLM)-induced fibrosis without detectable colonic side effects.*

(**a**) Composite histopathological scores of colonic tissue from each group. Scores were calculated as the sum of inflammation and erosion scores from hematoxylin and eosin (H&E)-stained sections. Nintedanib (NIDB) significantly increased colonic pathology, whereas ACF-2 did not induce detectable adverse effects. Representative colonic sections were selected from independent animals in each group. Bars represent mean ± SEM calculated from 10 mice (BLM and NIDB groups) and 14 mice (Sham and ACF-2 groups). Statistical analysis was performed using a one-tailed unpaired Student’s t-test (tails = 1, type = 3; unequal-variance assumption). * *p* < 0.05 vs. BLM; ## *p* < 0.01 vs. NIDB.

**Figure S1.**

**
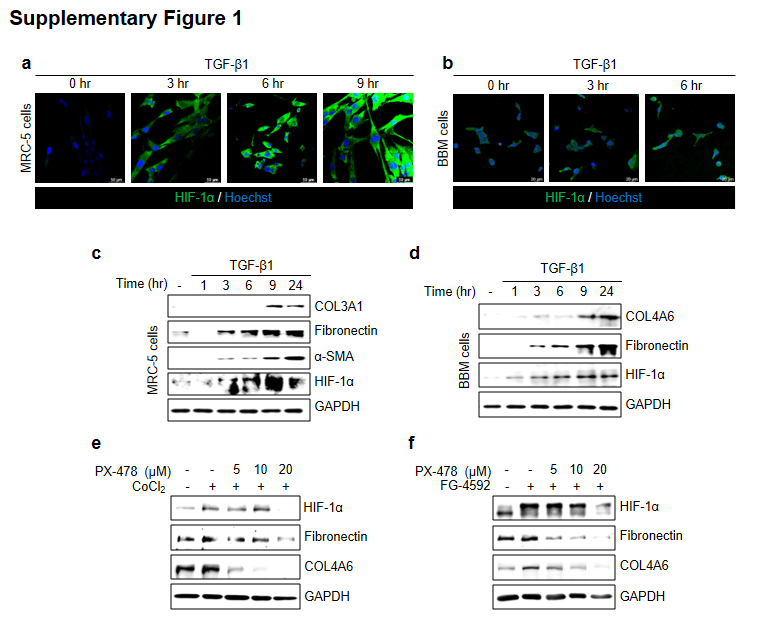
**

**Figure S2-1.**


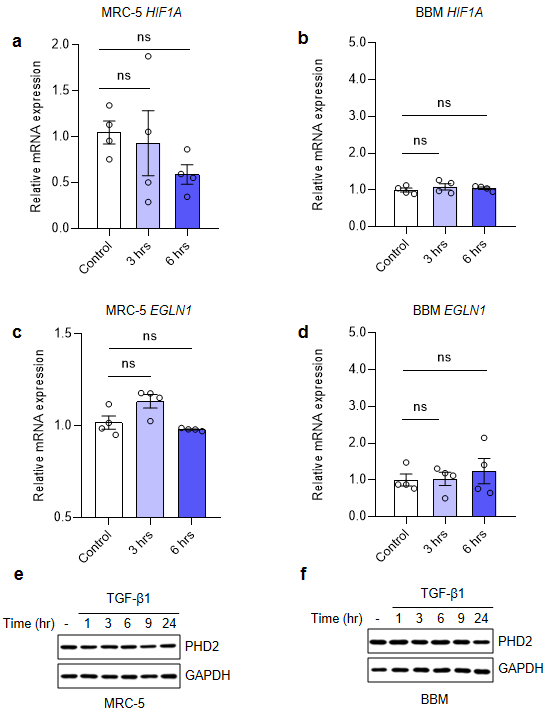


**Figure S2-2.**


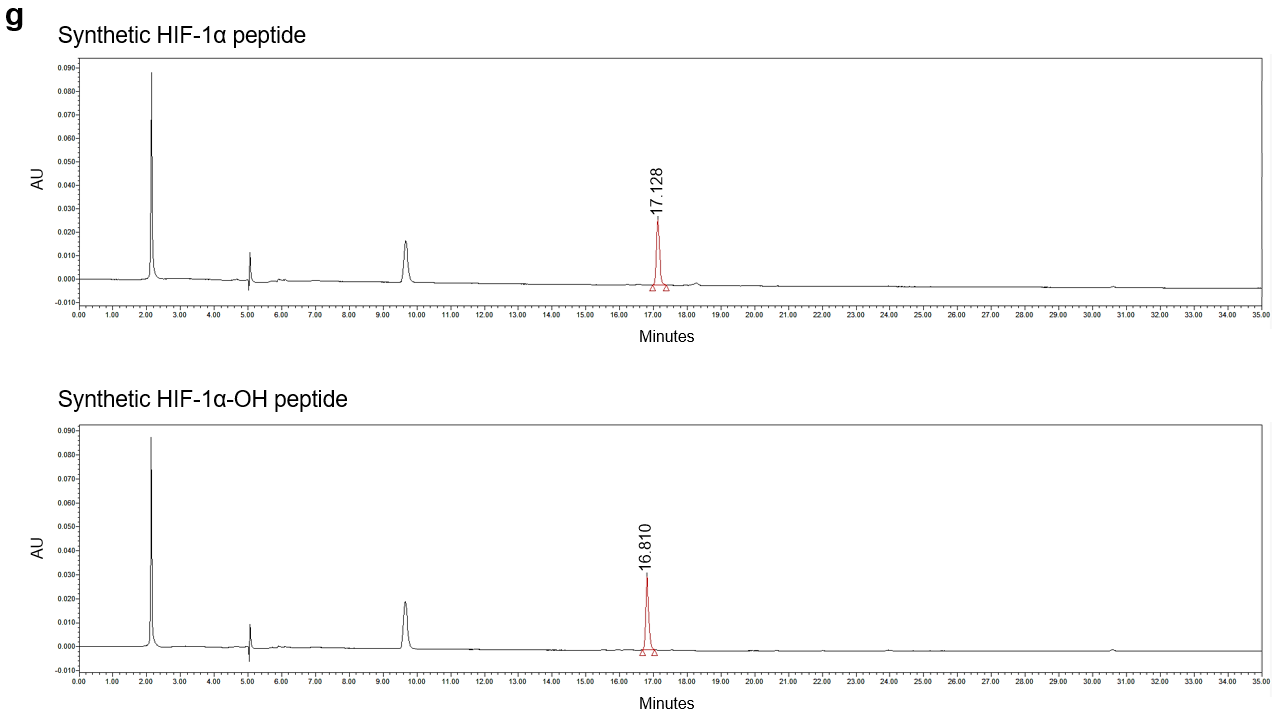


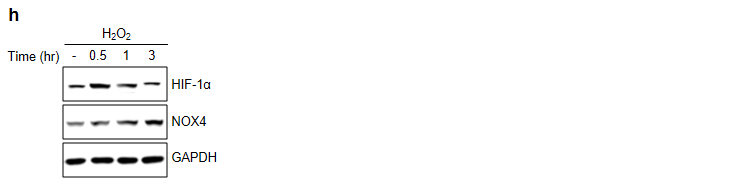


**Figure S3-1.**


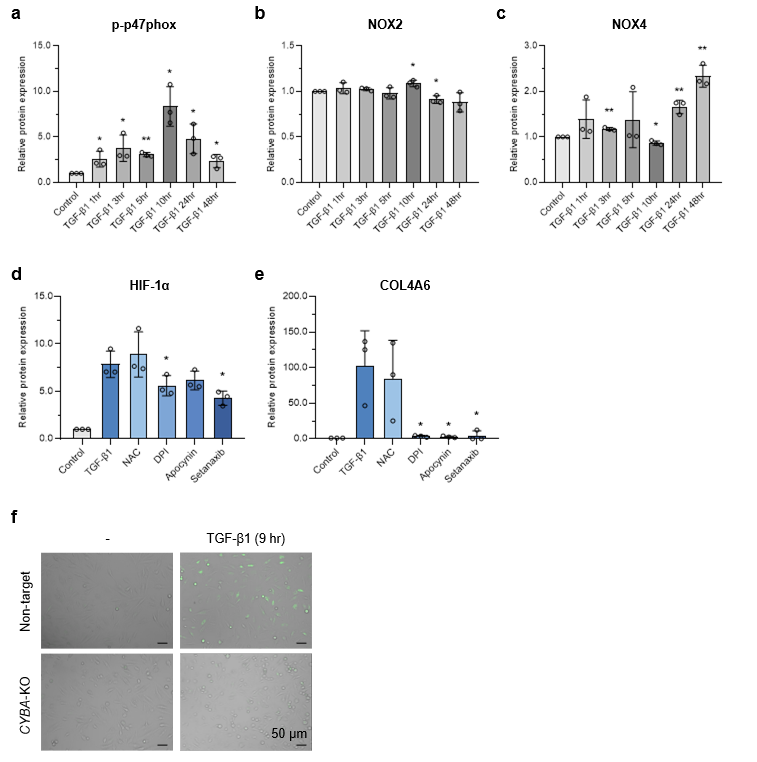


**Figure S3-2.**


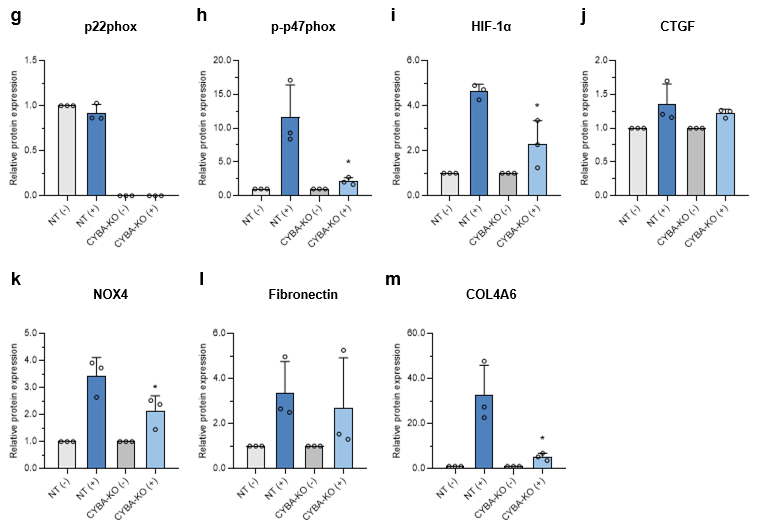


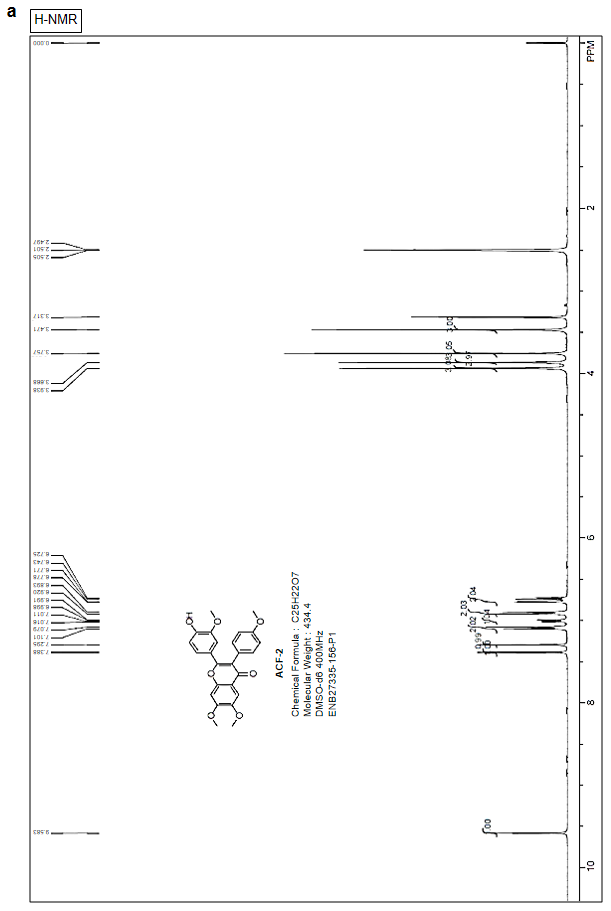
**Figure S4-1.**

**Figure S4-2.**


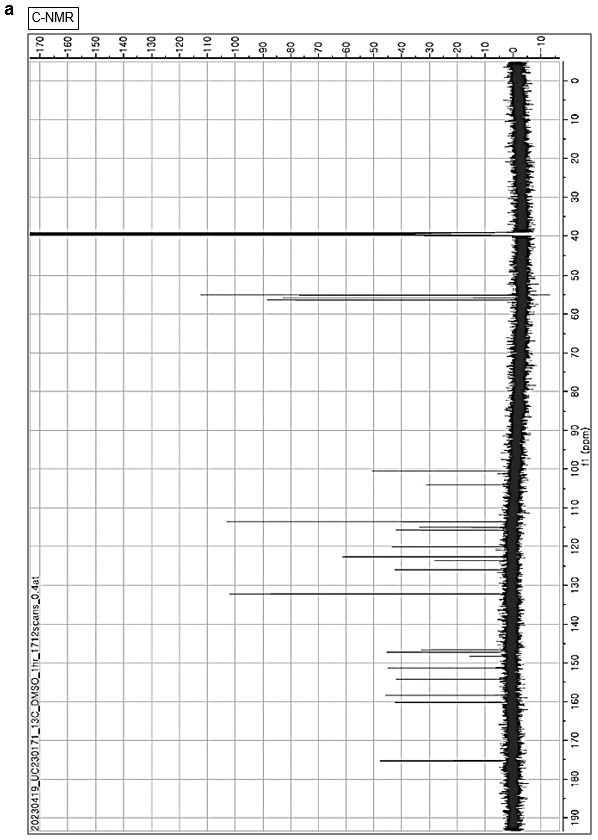
**Figure S4-3.**


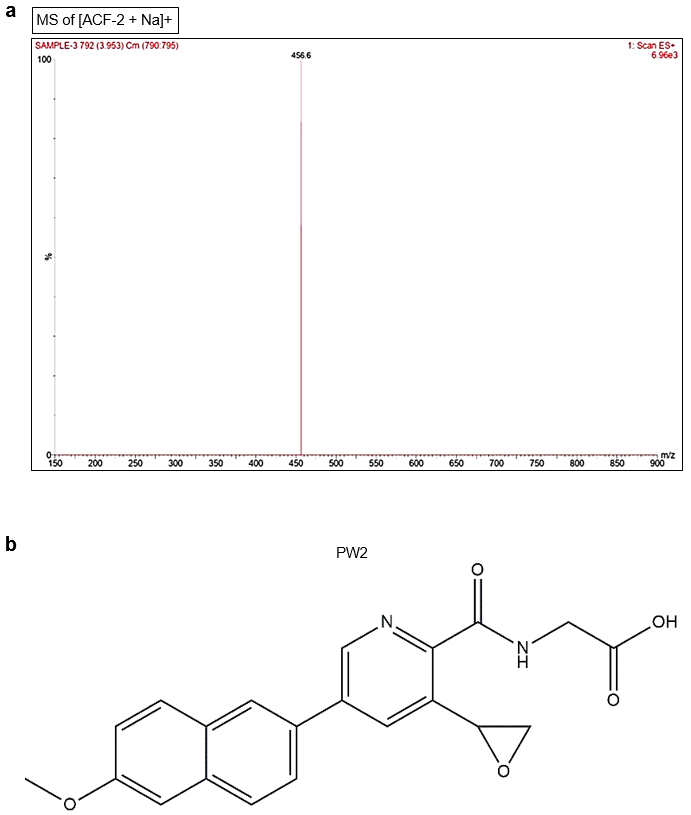


**Figure S4-4.**
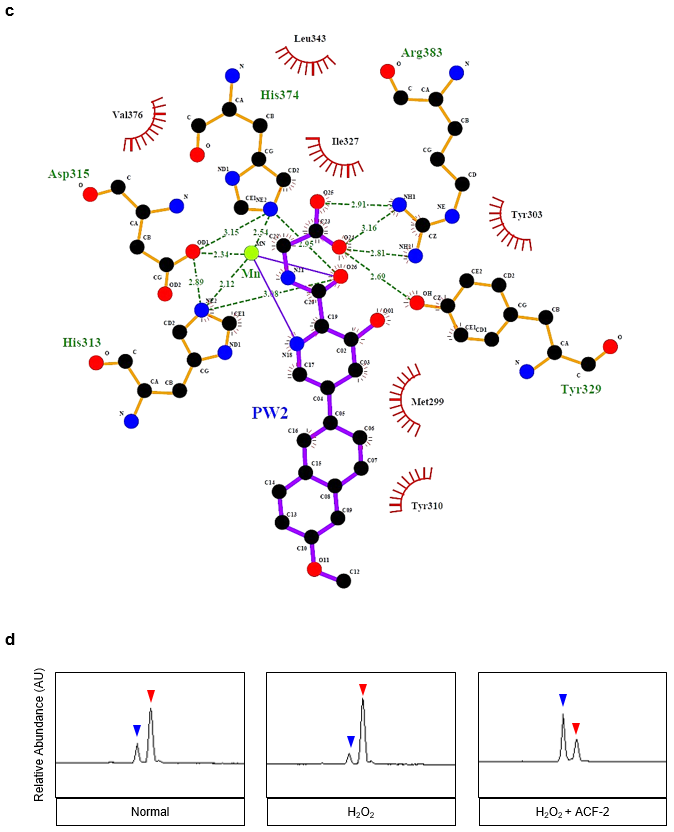


**Figure S5.**


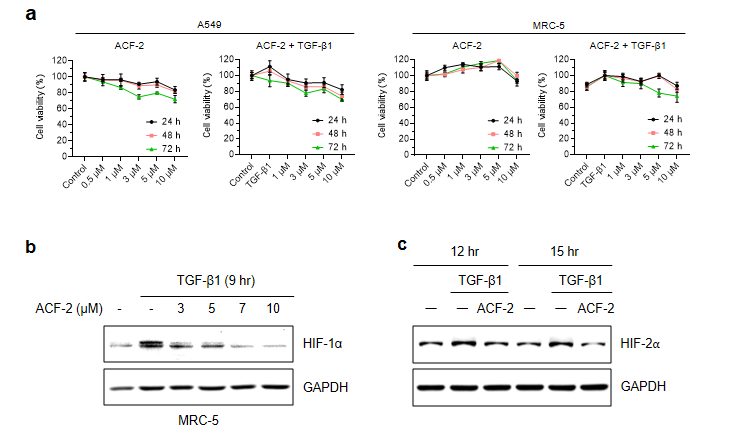


**Figure S6-1.**


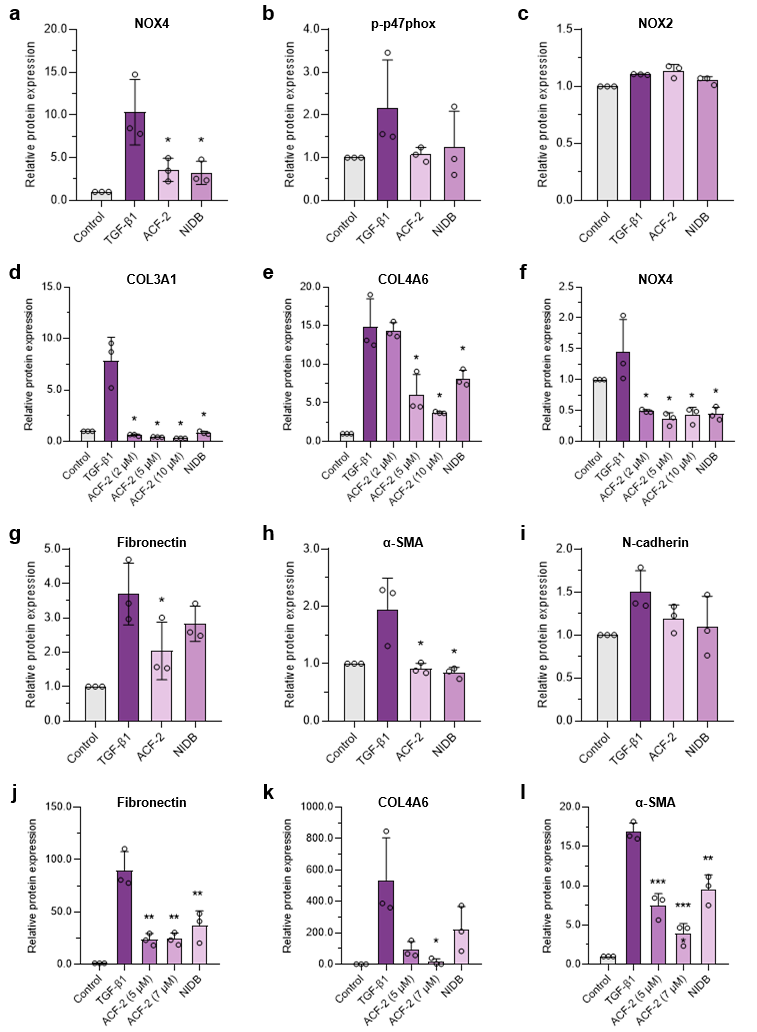


**Figure S6-2.**


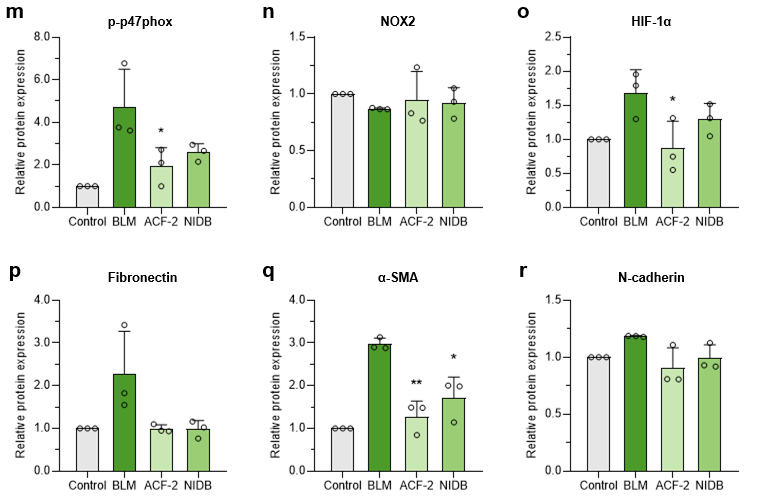


**Figure S7.**


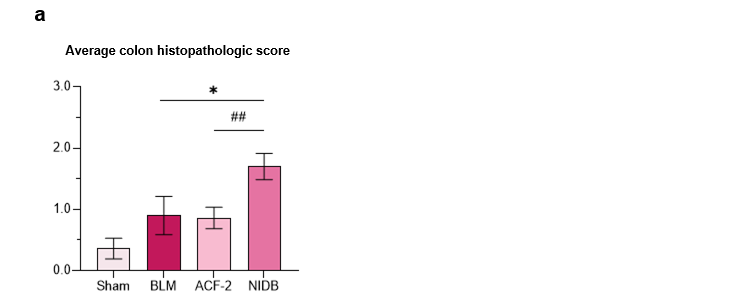


**② Document S2. Protocols & Materials (Table S1-S26)**

*Cell Lines and Culture*

Alveolar epithelial type II cells (A549), lung fibroblasts (MRC-5), and airway epithelial cells (BBM) were purchased from ATCC. A549 and BBM were maintained in high-glucose DMEM (4.5 g/L D-glucose), and MRC-5 was maintained in MEM (1 g/L D-glucose). All media were supplemented with 10% fetal bovine serum (FBS) and 1% penicillin–streptomycin, and cells were cultured at 37 °C in a humidified incubator with 5% CO₂ and ambient air (~18 % pericellular O₂). Except for MTT assays, where the serum concentration remained 10%, the FBS level was reduced to 2% for most biological assays and compound treatments and further reduced to 0.1% during siRNA transfection.

Table S1. Cell lines and basal culture media.

| **Materials** | **Manufacturer** | **Cat #** |
| --- | --- | --- |
| A549 | ATCC, USA | CCL-185 |
| MRC-5 | ATCC, USA | CCL-171 |
| BBM | ATCC, USA | CRL-9482 |
| DMEM | Welgene, South Korea | LM001-05 |
| MEM | Hyclone, Cytiva, USA | SH30024.01 |

*Combination Treatment*

Where indicated, TGF-β1, BLM, CoCl₂, FG-4592, or other agents were administered at the concentrations and durations specified in each experiment, simultaneously with ACF-2, PX-478, N-acetylcysteine (NAC), diphenyleneidonium chloride (DPI), apocynin, or Setanaxib. Control cells were treated with the corresponding vehicle (e.g., DMSO <0.01% or PBS), and the serum concentration was matched to that of the treatment groups. To inhibit protein degradation by the proteasome, MG-132 was used at 10 µM. VH-298, a pVHL antagonist that stabilizes hydroxylated HIF-1α, was applied at 50 µM. In experiments combining MG-132 with TGF-β1, FG-4592, or H₂O₂, the primary compound was applied for the indicated concentration and duration, followed by the addition of MG-132 10 min prior to the end of that incubation period. In experiments combining VH-298 with H₂O₂, VH-298 was applied using the same timing protocol as MG-132. To block de novo synthesis of protein, cells were first exposed to TGF‑β1 for 3 hr, after which cycloheximide was applied—either alone or in combination with ACF‑2—for the time periods specified in each experiment.

Table S2. Chemical reagents used for pharmacological or stress treatments

| **Materials** | **Manufacturer** | **Cat #** |
| --- | --- | --- |
| TGF-β1 | Gibco, Thermo Fisher Scientific, USA | PHG9214 |
| BLM sulfate | Tokyo Chemical Industry, Japan | B3972 |
| CoCl₂ | DAEJUNG, South Korea | 2570-4405 |
| FG-4592 | MedChemExpress, USA | HY-13426 |
| ACF-2 | Synthesized by,  and patent held by Achembio, South Korea | SH30024.01 |
| PX-478 | MedChemExpress, USA | HY-10231 |
| NAC | Sigma-Aldrich, Merck KGaA, Germany | A9165 |
| DPI | MedChemExpress, USA | HY-100965 |
| Apocynin | MedChemExpress, USA | HY-N0088 |
| Setanaxib | MedChemExpress, USA | HY-12298 |
| MG-132 | MedChemExpress, USA | HY-13259 |
| VH-298 | MedChemExpress, USA | HY-100947 |
| H₂O₂ | Sigma-Aldrich, Merck KGaA, Germany | H1009 |
| Cycloheximide | Sigma-Aldrich, Merck KGaA, Germany | 01810 |

*RNA Interference*

Transient RNA interference was conducted using AccuTarget™ Negative Control siRNA (5 nM) or AccuTarget™ custom-designed human siHIF-1α (5 nM) in combination with INTERFERin® reagent. On the day of transfection, the medium was replaced with 0.1% FBS, and cells were harvested 48 hr post-transfection.

Table S3. siRNA oligonucleotides and transfection reagent

| **Materials** | **Manufacturer** | **Cat #** |
| --- | --- | --- |
| AccuTarget™ Negative  Control siRNA | BIONEER, South Korea | SN-1021 |
| AccuTarget™ custom-  designed human siHIF-1α | BIONEER, South Korea | - |
| INTERFERin® reagent | Polyplus, France | 101000028 |

*Transient Transfection (pg-HIF-1α–EGFP)*

Transient transfection of A549 cells with pg-HIF-1α–EGFP was performed using jetOPTIMUS® according to the manufacturer’s protocol. Following 48 hr of transfection, the medium was replaced, and cells were treated with TGF-β1 at the specified concentration and duration before further analysis. Cell imaging was performed using an EVOS™ M7000 at 10×, 20×, or 40× magnification. GFP fluorescence intensity was measured using ImageJ. The mean fluorescence within the regions of interest (ROIs) was calculated after background subtraction, and values were normalized to the control condition

Table S4. Plasmid, imaging equipment and buffers for pg‑HIF‑1α–EGFP assays

| **Materials** | **Manufacturer** | **Cat #** |
| --- | --- | --- |
| pg-HIF-1α–EGFP | Addgene plasmid, USA | 87204 |
| EVOS™ M7000 | Thermo Fisher Scientific, USA | AMF7000HCA |
| RIPA buffer | Biosesang, South Korea | R2002-050-00 |
| ImageJ (version 1.53q) | National Institutes of Health, USA | _ |

*CRISPR/Cas9-Mediated CYBA Knockout in A549 Cells*

To generate *CYBA-*KO A549 cells, two distinct guide RNAs (gRNAs) targeting the human *CYBA* locus were constructed separately into the pSpCas9(BB)-2A-Puro (PX459) vector^1^. A549 cells were cultured in complete growth medium and transfected with each gRNA-containing PX459 construct using jetOPTIMUS, following the manufacturer’s instructions. 48 hr post-transfection, cells were subjected to puromycin selection (1 μg/mL). Once non-transfected control cells were eliminated, surviving cells harboring the *CYBA*-targeting gRNAs were isolated by endpoint dilution to obtain single-cell clones. Individual clones were expanded and screened by western blot analysis using an anti-p22phox antibody (p22phox is the protein encoded by *CYBA*). Clones exhibiting no detectable p22phox signal were classified as complete *CYBA* knockouts and were subsequently expanded for downstream experiments.

Table S5. Vector used for guide‑RNA construction.

| **Materials** | **Manufacturer** | **Cat #** |
| --- | --- | --- |
| pSpCas9(BB)-2A-Puro  (PX459) vector | Addgene plasmid, USA | 62988 |

Table S6. Guide‐RNA sequences targeting human *CYBA.*

| **Guide RNAs** | **sequence** |
| --- | --- |
| No.1 | 5′-ACAGAAGTACATGACCGCCG-3′ |
| No.2 | 5′-AGTAGGCACCAAAGTACCAC-3′ |

*Protein Extraction and Western Blot Analysis*

Cells were lysed in RIPA buffer supplemented with a Protease Inhibitor Cocktail. Protein concentrations were determined using the SMART™ BCA Protein Assay Kit. Equal amounts of protein were separated by SDS-PAGE and transferred onto nitrocellulose membranes. Membranes were blocked with 5% skim milk in TBST and incubated overnight at 4 °C with the primary antibodies indicated below. Membranes were then washed and incubated for 1 hr at room temperature with the appropriate secondary antibodies. Protein bands were visualized using the Davinch Western Imaging system according to the manufacturer’s instructions. Band intensities were quantified using ImageJ software after background subtraction. The signal intensity of each target protein was normalized to the corresponding GAPDH band, and values were further normalized to the control group (set to 1 for each independent experiment).

Table S7. Reagents and equipment for protein extraction and western blotting.

| **Materials** | **Manufacturer** | **Cat #** |
| --- | --- | --- |
| RIPA buffer | Biosesang, South Korea | R2002-050-00 |
| Protease Inhibitor  Cocktail | Sigma-Aldrich, Merck KGaA, Germany | S8820-2TAB |
| SMART™ BCA Protein  Assay Kit | INTRON, South Korea | 21071 |
| IgG (H&L) Anti-Mouse,  HRP | KOMA biotechnology, South Korea | K0211589 |
| IgG (H&L) Anti-Rabbit,  HRP | KOMA biotechnology, South Korea | K0211708 |
| Davinch Western Imaging system | Davinch-K, South Korea | CAS-400SM |
| ImageJ (version 1.53q) | National Institutes of Health, USA | _ |

Table S8. Primary antibodies used for western blot analysis.

| **Primary antibodies** | **Dilutions** | **Manufacturer** | **Cat #** |
| --- | --- | --- | --- |
| HIF-1α | 1:1,000 | Novus Biologicals, USA | NB100-449 |
| HIF-1α-OH | 1:2,000 | Cell Signaling Technology, USA | 3434S |
| PHD2 | 1:1,000 | Santa Cruz Biotechnology, USA | sc-271835 |
| HIF-2α | 1:1,000 | Cell Signaling Technology, USA | 71565S |
| COL3A1 | 1:1,000 | Santa Cruz Biotechnology, USA | sc-271249- |
| COL4A6 | 1:1,000 | Santa Cruz Biotechnology, USA | sc-398655 |
| Fibronectin | 1:2,000 | Novus Biologicals, USA | NBP2-66845 |
| α-SMA | 1:1,000 | Cell Signaling Technology, USA | 19245S |
| CTGF | 1:1,000 | Novus Biologicals, USA | NB100-724 |
| NOX2 | 1:1,000 | Novus Biologicals, USA | NBP2-67680 |
| NOX4 | 1:1,000 | Novus Biologicals, USA | NB110-58849 |
| p-p47phox | 1:2,500 | Sigma-Aldrich, Merck KGaA,  Germany | SAB4504721-100UG |
| p22phox | 1:1,000 | Cell Signaling Technology, USA | 37570S |
| E-cadherin | 1:1,000 | Cell Signaling Technology, USA | 3195S |
| N-cadherin | 1:1,000 | Santa Cruz Biotechnology, USA | sc-59987 |
| GAPDH | 1:3,000 | Santa Cruz Biotechnology, USA | sc-47724 |

*Migration Assay*

Cells were seeded at 3 × 10⁴ cells per well in 200 µL of medium in a 96-well plate. Before treating with the test compounds, a vertical scratch (wound) was made across each well using the BioTek AutoScratch™ Accessory & Supplies. Detached cells were removed by washing with PBS, and fresh medium containing the test compound(s) was added. Cells were then incubated at 37 °C in a humidified atmosphere of 5% CO₂ for 48 hr, with real-time monitoring of cell migration using a BioTek Cytation 5 Cell Imaging Multi-Mode Reader. Wound closure ratio (%) was calculated based on the horizontal distance (µm) of the wound edge measured by the Cytation 5 software. Mean closure values were analyzed using Student’s t-test (tails =1, type = 3) to determine statistical significance.

Table S9. Consumables and instruments for scratch‑migration assay.

| **Materials** | **Manufacturer** | **Cat #** |
| --- | --- | --- |
| 96-well plate | Corning, USA | 3598 |
| BioTek AutoScratch™  Accessory & Supplies | Agilent Technologies, USA | - |
| BioTek Cytation 5  Cell Imaging Multi-Mode Reader | Agilent Technologies, USA | - |

*RNA Isolation and RT-qPCR*

Total RNA was extracted from both cultured cells and mouse lung tissue using the AccuPrep® Universal RNA Extraction Kit. RNA concentration and purity were assessed with a SpectraMax® QuickDrop™ UV-Vis Spectrophotometer. cDNA was synthesized with the DiaStar™ RT Kit using random hexamers. RT-qPCR was performed using iTaq™ Universal SYBR® Green Supermix on a StepOnePlus™ Real-Time PCR System. The primers used in this study were synthesized by COSMO GENETECH.

Table S10. Reagents and instruments for RNA isolation and RT‑qPCR.

| **Materials** | **Manufacturer** | **Cat #** |
| --- | --- | --- |
| AccuPrep® Universal  RNA Extraction Kit | BIONEER, South Korea | K-3140 |
| SpectraMax®  QuickDrop™ UV-Vis  Spectrophotometer | Molecular Devices, USA | - |
| DiaStar™ RT Kit | Solgent, South Korea | DR22-R10K |
| Random hexamers | Invitrogen™, Thermo Fisher Scientific, USA | N8080127 |
| iTaq™ Universal SYBR® Green Supermix | Bio-Rad Laboratories, USA | 1725121 |
| StepOnePlus™  Real-Time PCR System | Applied Biosystems, Roche Diagnostics,  Germany | - |
| RT-qPCR primers | Synthesized by COSMO GENETECH,  South Korea | - |

Table S11. RT‑qPCR primer sequences (human & mouse).

|  | **Gene names** | **sequence** |
| --- | --- | --- |
| For human gene  expression analysis | *HIF1A* | forward: 5′-CCA CAG GAC AGT ACA GGA TG-3′  reverse: 5′-TCA AGT CGT GCT GAA TAA TAC C-3′ |
|  | *EGLN1* | forward: 5′-GTG CCG TGC ATG AAC AAG C-3′  reverse: 5′-CAG GTG ATC TTA TCG CCT CGG-3′ |
|  | *18S rRNA,*  as internal control | forward: 5′-GGC CCT GTA ATT GGA ATG AGT C-3′  reverse: 5′-CCA AGA TCC AAC TAC GAG CTT-3′ |
| For mouse gene  expression analysis | *Tgfb1* | forward: 5′-CGA CCA GGA AAT GGG CTA AA-3′  reverse: 5′-CAA CCA GAC AGT GGT CCT AAT C-3′ |
|  | *Col1a1* | forward: 5′-CAC CCT TCC AAG TCC TCA TTT-3′  reverse: 5′-CTT ACT CGG TGT CCC TTC ATT C-3′ |
|  | *Fn1* | forward: 5′-GGG AGG AAG AAG ACA GGT AAA G-3′ reverse: 5′-GCC ACT CTC TGC TAG GGA AAT AA-3′ |
|  | *Acta2* | forward: 5′-GGC TGA GAT TTG CTT TCT GAT G-3′  reverse: 5′-GTG TGC TTG GGT GTG TTT ATG-3′ |
|  | *Actb,*  as internal control | forward: 5′-GAG GTA TCC TGA CCC TGA AGT A-3′  reverse: 5′-CAC ACG CAG CTC ATT GTA GA-3′ |

*MTT Assay*

Cells were seeded at 8 × 10³ cells per well in 200 µL of medium in a 96-well plate. The next day, the medium was replaced with medium containing the test compound(s) at the desired concentration(s) and incubated for the specified duration. After the incubation, the medium was discarded and replaced with 0.5 mg/mL Thiazolyl Blue Tetrazolium Bromide in PBS. Following a 4 h incubation at 37 °C, the formazan crystals were dissolved in DMSO, and absorbance was measured at 570 nm using a SUNRISE™ microplate reader. The relative cell viability was calculated based on the optical density values.

Table S12. Reagents and microplate reader for MTT viability assay.

| **Materials** | **Manufacturer** | **Cat #** |
| --- | --- | --- |
| Thiazolyl Blue  Tetrazolium Bromide | Sigma-Aldrich, Merck KGaA,  Germany | M2128-1G |
| DMSO | LPS SOLUTION, South Korea | DMSO100 |
| SUNRISE™ microplate  reader | TECAN, Switzerland | 30087502 |

*ROS Staining*

CellROX™ Deep Red: Cells were seeded at 3 × 10⁴ cells per well in 1 mL of medium on Cell Culture Slide I. The next day, the medium was replaced with fresh medium containing the test compound(s) and incubated for the indicated period. Subsequently, CellROX™ Deep Red Reagent was added to a final concentration of 5 µM and incubated for 30 min at 37 °C. Hoechst 33342 (0.5 µg/mL) was included during the final 10 min. After discarding the medium and washing once with PBS, fluorescence was detected in the Cy5 channel using an EVOS™ M7000 imaging system.

Dihydrorhodamine (DHR)123: Cells were similarly seeded at 3 × 10⁴ cells per well in 1 mL of medium on Cell Culture Slide I. After the desired treatments and incubation times, the medium was removed, cells were washed with PBS, and 10 µM Dihydrorhodamine 123 in PBS was applied for 30 min at 37 °C. Hoechst 33342 (0.5 µg/mL) was added for the final 10 min. Cells were then washed in PBS and imaged with the EVOS™ M7000 system in the Cy2 channel.

CM-H₂DCFDA: Cells (2 × 10⁵) were plated in 2 mL of medium per well in a 6-well plate. After treatment for the indicated times, the medium was removed, and cells were washed once with PBS. CM-H₂DCFDA (10 µM) in PBS was added for 30 min at 37 °C, followed by a 10 min equilibration in PBS. Cells were then detached with trypsin for 1 min, neutralized with FBS, pelleted by centrifugation, and resuspended in PBS. Fluorescence was measured using a CytoFLEX LX flow cytometer (excitation/emission: 485/520 nm).

Table S13a. Materials for CellROX™ Deep Red ROS staining.

| **Materials** | **Manufacturer** | **Cat #** |
| --- | --- | --- |
| Cell Culture Slide I  (4-well, Clear) | SPL Life Sciences, South Korea | 30104 |
| CellROX™ Deep Red  Reagent | Invitrogen™, Thermo Fisher Scientific, USA | C10422 |
| Hoechst 33342 | Cell Signaling Technology, USA | 4082S |

Table S13b. Materials for DHR123 ROS staining.

| **Materials** | **Manufacturer** | **Cat #** |
| --- | --- | --- |
| DHR123 | Sigma-Aldrich, Merck KGaA,  Germany | 309825-5MG |

Table S13c. Materials for CM‑H₂DCFDA flow‑cytometric ROS assay.

| **Materials** | **Manufacturer** | **Cat #** |
| --- | --- | --- |
| 6-well plate | SPL Life Sciences, South Korea | 30006 |
| CM-H₂DCFDA | Invitrogen™, Thermo Fisher Scientific, USA | C6827 |
| Trypsin | Gibco, Thermo Fisher Scientific, USA | 25200056 |
| CytoFLEX LX  flow cytometer | Beckman Coulter, USA | - |

*DPPH Assay*

ACF-2 (7 and 10 µM, 100 µL/well) was loaded into a 96-well plate (non-treated surface). Catechin and NAC were similarly prepared at 10 µM in 100 µL. DPPH (2,2-diphenyl-1-picrylhydrazyl) at 100 µM in methanol was then added (100 µL/well) and allowed to react for 30 min in the dark. Absorbance was measured at 517 nm on SUNRISE™ microplate reader, and radical scavenging activity was evaluated based on the optical density readings.

Table S14. Reagents and positive controls for the DPPH radical‑scavenging assay.

| **Materials** | **Manufacturer** | **Cat #** |
| --- | --- | --- |
| Catechin | MedChemExpress, USA | HY-N0898 |
| NAC | Sigma-Aldrich, Merck KGaA,  Germany | A9165 |
| DPPH | MedChemExpress, USA | HY-112053 |

*Surface plasmon resonance (SPR)*

The cDNA encoding human PHD2 (Pro_181_–Phe_426_, tPHD2) was subcloned into a pET-28a vector containing a TEV cleavage site. The recombinant His-tagged protein was then expressed in BL21(DE3) *E. coli* and purified via ÄKTA go^TM^ FPLC. Interaction studies between ACF-2 and tPHD2 were conducted on an iMSPR-ProX instrument at room temperature. The running buffer comprised 10 mM phosphate, 140 mM NaCl, 2.7 mM KCl, 0.005% Tween 20, and 1% DMSO (pH 7.4). tPHD2 was immobilized on an HC1000 chip via amine coupling and avidin–biotin capture, achieving approximately 8,133 response units. ACF-2 was injected over the sensor surface in ascending concentrations (0.78, 1.56, 3.13, 6.25, 12.5, 25 µM) at a flow rate of 50 µL/min. A 3 M NaCl solution was then introduced at the same flow rate to dissociate ACF-2 from the chip. A one-to-one binding model, implying a single binding site for one ligand molecule, was used to derive the association (*k*_a_) and dissociation (*k*_d_) rate constants, and the equilibrium dissociation constant (K*_D_*) was computed as *k*_d_/*k*_a_. Curve fitting and data processing were performed with iMSPR analysis software.

Table S15. FPLC system for tPHD2 purification and SPR instruments, chips, and software.

| **Materials** | **Manufacturer** | **Cat #** |
| --- | --- | --- |
| ÄKTA go^TM^ FPLC | Cytiva, USA | - |
| iMSPR-ProX instrument | iCLUEBIO, South Korea | - |
| HC1000 chip | iCLUEBIO, South Korea | - |
| iMSPR analysis software | Tracedrawer; iCLUEBIO, South Korea | - |

*In vitro PHD2 assay*

A reaction mixture (30 µL total volume) containing 300 µM HIF peptide, 1 µM recombinant PHD2, 20 mM Tris-HCl (pH 7.5), 1 mM DTT, 300 µM ascorbic acid, 5 mM KCl, 1.5 mM MgCl_2_, 50 µM Fe(SO_4_)·6H_2_O, and 300 µM α-ketoglutarate was incubated at 30 °C for 2 hr. The reaction was terminated by adding an equal volume (30 µL) of DMSO. Product analysis was carried out on Waters 2998 HPLC system equipped with a photodiode array detector and an XBridge™ BEH C18 column (4.6 × 250 mm, 5 µm), detecting at 280 nm. A hydroxylated HIF peptide served as a reference standard.

Table S16. Peptides, enzyme and HPLC column for *in vitro* PHD2 assay.

| **Materials** | **Manufacturer** | **Cat #** |
| --- | --- | --- |
| HIF peptide (Biotin-DLDLEALAPYIPADDDFQL) | Custom-synthesized by  PEPTRON, South Korea | - |
| Hydroxylated HIF peptide (Biotin-DLDLEALA-Hyp-YIPADDDFQL) |  |  |
| PHD2 | ActiveMotif, Japan | 81065 |
| Waters 2998 HPLC  system | Waters Corporation, USA | - |
| iTaq™ Universal SYBR® Green Supermix | Bio-Rad Laboratories, USA | 1725121 |
| StepOnePlus™  Real-Time PCR System | Applied Biosystems, Roche Diagnostics,  Germany | - |
| RT-qPCR primers | Synthesized by COSMO GENETECH,  South Korea | - |

*In vitro Pharmacokinetics*

Metabolic Stability*:* To evaluate metabolic stability of ACF-2, pooled human liver microsomes (HLMs, Xenotech H0630) and ACF-2 (1 µM) were pre-incubated. To initiate the reaction, NADPH generating system was added to the incubation samples and further incubated for specific time points (0, 5, 15, 30, 45, and 60 min). Then, sample aliquots were taken and terminated with cold acetonitrile. After centrifugation, supernatants were analyzed using liquid chromatography-tandem mass spectrometry (LC-MS/MS) to determine the concentration of ACF-2 in sample. Separation of ACF-2 was performed on a Kinetex XB-C18 column (100 × 2.1 mm, 2.6 µm, 100Å) with an isocratic mobile phase consisting of acetonitrile and water (55/45, v/v) containing 0.1% formic acid. Detection of the ACF-2 was performed by monitoring the transitions of m/z 435 > 437.

Cytochrome P450 Inhibition Assay*:* The inhibitory potential of ACF-2 was determined with cytochrome P450 assays in the presence and absence of ACF-2 (final concentrations of 0–50 µM) using HLMs. All experiments were performed in triplicate. Phenacetin O-deethylase, coumarin hydroxylase, bupropion hydroxylase, amodiaquine N-deethylase, tolbutamide 4-hydroxylase, S-mephenytoin hydroxylase, dextromethorphan O-demethylase, chlorozoxazone 6-hydroxylase, and midazolam 1’-hydroxylase activities were determined as probe activities for CYP1A2, CYP2A6, CYP2B6, CYP2C8, CYP2C9, CYP2C19, CYP2D6, CYP2E1, and CYP3A, respectively, using cocktail incubation and tandem mass spectrometry, as described previously^2,3^.

Table S17. Reagents and LC–MS/MS setup for *in vitro* pharmacokinetic studies.

| **Materials** | **Manufacturer** | **Cat #** |
| --- | --- | --- |
| Microsomes  (HLMs, H0630) | XenoTech (Lenexa, USA) | - |
| Liquid chromatography-tandem mass spectrometry | Shimadzu, Japan | LCMS-8060 |
| Kinetex XB-C18 column | Phenomenex Torrance, USA | - |

*In silico 3D binding*

Three-dimensional (3D) structures of PHD2 are available in the Protein Data Bank (PDB), totaling 24 after excluding redundant entries. Among these, PDB ID 6yvt was chosen because it encompasses most PHD2 residues and includes a ligand structurally similar to ACF-2^4^. This ligand, 2-[[5-(6-methoxynaphthalen-2-yl)-3-oxidanyl-pyridin-2-yl]carbonylamino]ethanoic acid (named PW2), is found in a crystallographic structure composed of heteropentameric polypeptides. For *in silico* docking, Chain A (L188–L404; 217 residues) was selected. Its solution structure was modeled using molecular dynamics (MD) simulations in Sybyl 7.3 (Tripos, St. Louis, MO, USA). The docking site was identified using AutoDock Tools (ADT; Scripps Research Institute, La Jolla, CA, USA)^5^, with the grid box centered at x = 22.361, y = −10.611, and z = −71.083, each dimension set to 24 × 24 × 24. All subsequent docking was carried out via ADT. The protein–ligand complexes were generated using Chimera^6^, and binding interactions were evaluated with LigPlot^7^. Final 3D images of the complexes were produced in PyMOL (Schrödinger, LLC).

Table S18. Software and toolkits for *in silico* 3D docking of PHD2 ligands.

| **Materials** | **Manufacturer** | **Cat #** |
| --- | --- | --- |
| Sybyl 7.3 | Tripos, USA | - |
| AutoDock Tools | Scripps Research Institute, USA | - |

*Computational Prediction of ADME Properties*

ADME-related parameters for each compound were predicted using SwissADME^8^ with default settings. SMILES structures were generated for ACF-2 and nintedanib (NIDB), then uploaded into the SwissADME interface. From these inputs, SwissADME computed physicochemical descriptors as well as pharmacokinetic estimates, including consensus lipophilicity (LogP_o/w), solubility, gastrointestinal absorption, blood–brain barrier penetration, interactions with P‑glycoprotein (Pgp) and cytochrome P450 isoforms, and skin permeability (LogKp). Collectively, these data provided a comprehensive assessment of each compound’s drug-likeness profile.

*Nuclear Magnetic Resonance (NMR)*

NMR spectra to identify the structure of ACF-2 were recorded on a Bruker Avance III 400 (9.4 Tesla, Bruker gmbh, Karlsruhe, Germany). ACF-2 was dissolved in D₂O or DMSO-d₆ at a final concentration of ~50 mM and transferred to a standard 5 mm NMR tube. Spectra were acquired at room temperature and analyzed with SPARKY 3 (developed by T. D. Goddard and D. G. Kneller, University of California, San Francisco). The chemical shifts were referenced to tetramethylsilane. The detailed procedures followed to the methods reported previously^9^.

Table S19. NMR spectrometer and analysis software for compound identification.

| **Materials** | **Manufacturer** | **Cat #** |
| --- | --- | --- |
| Avance III™ HD 400 MHz NMR spectrometer | Bruker BioSpin GmbH, Germany | - |

*Mice Experiment*

All animal studies were approved by the Institutional Animal Care and Use Committee of Jeju National University (Approval ID: 2024-0058) and conducted in compliance with institutional guidelines. Male ICR mice (6 weeks old, average body weight 27 g) were purchased from SAMTAKO (South Korea) and housed under controlled temperature (22 ± 2 °C) and humidity (0.0 ± 15.0 %) on a 12 hr light–dark cycle with free access to food and water. Pulmonary fibrosis was induced by a single intratracheal instillation of BLM (30 mg/kg body weight; BLM sulfate) under avertin anesthesia (2,2,2-tribromoethanol). Control mice received an equivalent volume of distilled water (DW). Beginning 7 days after instillation, mice in the ACF-2 group received an intraperitoneal injection of ACF-2 (10 mg/kg every 2 days in 95% PBS and 5% DMSO, total volume 400 µL) for 2 weeks, while the NIDB group was given NIDB (60 mg/kg/day dissolved in DW, average daily intake of 7 mL/mouse). The sham and BLM groups were injected with 95% PBS and 5% DMSO. Body weight and clinical signs were monitored daily. After drug administration, the mice were euthanized humanely, and lung tissues were collected for histopathology, western blot, and RT-qPCR analyses.

Table S20. Animal model materials and anesthesia for BLM fibrosis study.

| **Materials** | **Manufacturer** | **Cat #** |
| --- | --- | --- |
| Male ICR mice | SAMTAKO, South Korea | - |
| BLM sulfate | Tokyo Chemical Industry, Japan | B3972 |
| Avertin | Sigma-Aldrich, Merck KGaA,  Germany | T48402 |

*Histological Examinations*

Lung tissue: Left lung lobes were fixed in 4% paraformaldehyde for 24 h, dehydrated, and embedded in paraffin. Sections of 4 µm thickness were prepared, deparaffinized with xylene, and rehydrated in a graded ethanol series. H&E staining was performed using hematoxylin and eosin solutions, respectively. The severity of fibrosis was graded according to the Ashcroft scale (1–8), as evaluated by a pathologist blindly. Representative H&E images were chosen to illustrate the most characteristic pathology. Masson’s trichrome staining was conducted using a commercial kit.

Colon tissue: Colons were harvested, flushed with 1xPBS, and prepared using the swiss-roll method before embedding in paraffin. Sections of 4 µm thickness were subjected to H&E staining as described above. Colonic injury was evaluated by two independent parameters: (i) inflammation score (0–3) and (ii) erosion score (0–3). A composite histological score was then calculated as the sum of inflammation and erosion scores for each sample.

Table S21. Histology reagents and staining kit.

| **Materials** | **Manufacturer** | **Cat #** |
| --- | --- | --- |
| Hematoxylin | Biognost, Croatia | Hemh-OT-1L |
| Eosin | Mirax, South Korea | 3610MIRA01 |
| Masson’s trichrome  staining kit | Biognost, Croatia | MST-K-500 |

*Immunohistochemistry*

Paraffin-embedded lung sections (4 µm) were deparaffinized and rehydrated using standard xylene/alcohol methods, followed by incubation with hydrogen peroxide (room temperature, 5 min) and trypsin (37 °C, 45 min) for antigen retrieval. After blocking with 1% bovine serum albumin, slides were incubated overnight at 4 °C with the primary antibodies indicated below. Dako Real Envision™ staining solution was then applied at 20 °C for 60 min with the respective anti-rabbit secondary antibodies, following the manufacturer’s guidelines. Sections were counterstained with Mayer’s hematoxylin, dehydrated, and scanned on a Aperio CS2 slide scanner at x20 magnification.

Table S22. Reagents and instruments for immunohistochemistry.

| **Materials** | **Manufacturer** | **Cat #** |
| --- | --- | --- |
| Dako LSAB2 System  -HRP Kit | Agilent, USA | K0675 |
| Trypsin | Sigma-Aldrich, Merck KGaA,  Germany | T7409-1G |
| Dako Real Envision™  staining solution | Agilent, USA | K500711-2 |
| Mayer’s hematoxylin | Sigma-Aldrich, Merck KGaA,  Germany | MHS32 |
| Aperio CS2 slide scanner | Leica Biosystems, Germany |  |

Table S23. Primary antibodies for immunohistochemistry.

| **Primary antibodies** | **Dilutions** | **Manufacturer** | **Cat #** |
| --- | --- | --- | --- |
| HIF-1α | 1:200 | Novus Biologicals, USA | NB100-449 |
| CTGF | 1:200 | Novus Biologicals, USA | NB100-724 |
| NOX4 | 1:200 | Boster Bio, USA | M00403 |
| α-SMA | 1:200 | Cell Signaling Technology, USA | 19245S |

*Immunofluorescence*

Cells were fixed in 4% paraformaldehyde, permeabilized in 1% Triton X-100, and blocked with 5% BSA for 30 min at room temperature. Samples were then incubated overnight at 4 °C with the primary antibodies indicated below. After washing with PBS, samples were incubated with fluorophore-conjugated secondary antibodies, Alexa Fluor® 488 or 594 (1:1,000) for 1 h at room temperature. Nuclei were counterstained with Hoechst 33342 (0.5 µg/mL). Control staining was performed by omitting the primary antibodies. Stained cells or tissue sections were examined using a STELLARIS 5 Confocal Microscope at 40× magnification. Where applicable, mean fluorescence intensities within defined ROIs were quantified using ImageJ software, after background subtraction and normalized to the corresponding control condition. For mouse tissues, paraffin sections (4 µm) were deparaffinized in xylene and rehydrated through an ethanol gradient. Antigen retrieval was carried out in citrate buffer (pH 6.0) in a pressure cooker for 15 min. Slides were rinsed in dH₂O, blocked with normal horse serum for 1.5 hr at room temperature, and, if needed, treated with BD Pharmingen™ Purified Rat Anti-Mouse CD16/CD32 (5 µg/mL) for 10 min. Primary antibodies were applied overnight at 4 °C. The following day, Alexa Fluor–conjugated secondary antibodies (1:500) were incubated for 1 hr at room temperature, and nuclei were counterstained with Hoechst 33342 for 5 min. Images were obtained with an Olympus BX51 microscope at 40× magnification.

Table S24. Reagents and microscopes for cell & tissue immunofluorescence.

|  | **Materials** | **Manufacturer** | **Cat #** |
| --- | --- | --- | --- |
| For the cells | 4% paraformaldehyde | Sigma-Aldrich, Merck KGaA,  Germany | 158127 |
|  | Triton X-100 | LPS SOLUTION, South Korea | TRX-01 |
|  | Alexa Fluor® 488 | Cell Signaling Technology, USA | 4412S |
|  | Alexa Fluor® 594 | Cell Signaling Technology, USA | 8890S |
|  | Hoechst 33342 | Cell Signaling Technology, USA | 4082S |
|  | STELLARIS 5  Confocal  Microscope | Leica Microsystems, Germany | - |
|  | ImageJ (version 1.53q) | National Institutes of Health, USA | _ |
| For the tissue sections | Normal horse serum | Vector Laboratories, USA | MP-7402 |
|  | BD Pharmingen™ Purified  Rat Anti-Mouse  CD16/CD32  (BD Fc Block™) | BD Biosciences, USA | 553142 |
|  | Anti-rabbit IgG (H+L), F(ab')2 Fragment (Alexa Fluor® 488 Conjugate) | Cell Signaling Technology, USA | 4412S |
|  | Anti-mouse IgG (H+L),  F(ab')2 Fragment (Alexa  Fluor® 594 Conjugate) | Cell Signaling Technology, USA | 8890S |
|  | Donkey anti-Rat IgG (H+L)  Highly Cross-Adsorbed  Secondary Antibody,  Alexa Fluor™ 594 | Invitrogen™,  Thermo Fisher Scientific, USA | A-21209 |
|  | Donkey anti-Goat IgG (H+L) Cross-Adsorbed Secondary Antibody, Alexa Fluor™ 647 | Invitrogen™,  Thermo Fisher Scientific, USA | A-21447 |
|  | Hoechst 33342 | Cell Signaling Technology, USA | 4082S |
|  | Olympus BX51  microscope | Olympus Corporation, Japan | - |

Table S25. Primary antibodies for cell & tissue immunofluorescence.

|  | **Primary antibodies** | **Dilutions** | **Manufacturer** | **Cat #** |
| --- | --- | --- | --- | --- |
| For the cells | HIF-1α | 1:200 | Novus Biologicals, USA | NB100-449 |
|  | NOX4 | 1:200 | Boster Bio, USA | M00403 |
|  | PHD2 | 1:200 | Santa Cruz Biotechnology, USA | sc-271835 |
| For the tissue sections | HIF-1α | 1:50 | R&D Systems, USA | AF1935 |
|  | SFTPC | 1:200 | Abnova, Taiwan | H00006440-M01 |
|  | FAP | 1:150 | Novus Biologicals, USA | MAB9727-100 |
|  | NOX4 | 1:200 | Boster Bio, USA | M00403 |

*Statistical Analysis*

All statistical analyses were conducted using Microsoft Excel and GraphPad Prism 8. In preliminary exploratory experiments (e.g., compound screening and assay optimization), two-tailed unpaired Student’s *t*-tests were applied to evaluate general differences between groups. For hypothesis-driven experiments described in the main figures, where the direction of change (e.g., reduction in ROS, HIF-1α stabilization, or fibrotic markers) was predefined based on the mechanistic expectation of ACF-2, one-tailed unpaired Student’s *t*-tests were performed using the Excel *T.TEST* function (tails = 1, type = 3; unequal variance assumption). GFP fluorescence intensity data were analyzed by one-way ANOVA followed by Sidak’s multiple-comparisons test to determine group differences. All data are expressed as mean ± standard error, and exact p-values are provided in the figure legends where applicable. The number of independent biological replicates (n) for each experiment is indicated in the respective figure legends. Technical replicates refer to repeated measurements from the same culture, whereas biological replicates refer to independently cultured samples obtained on different days. Representative images were selected from at least two independent experiments, each containing ≥ 5 random fields of view.

Table S26. Software for statistical analysis.

| **Materials** | **Manufacturer** | **Cat #** |
| --- | --- | --- |
| Microsoft Excel | Microsoft Corporation, USA |  |
| GraphPad Prism 8.0 | GraphPad Software, USA |  |

**References**

1. Ran, F.A., Hsu, P.D., Wright, J., Agarwala, V., Scott, D.A., and Zhang, F. (2013). Genome engineering using the CRISPR-Cas9 system. Nat Protoc *8*, 2281-2308. 10.1038/nprot.2013.143.

2. Kim, M.J., Kim, H., Cha, I.J., Park, J.S., Shon, J.H., Liu, K.H., and Shin, J.G. (2005). High-throughput screening of inhibitory potential of nine cytochrome P450 enzymes in vitro using liquid chromatography/tandem mass spectrometry. Rapid Commun Mass Spectrom *19*, 2651-2658. 10.1002/rcm.2110.

3. Kim, H.J., Lee, H., Ji, H.K., Lee, T., and Liu, K.H. (2019). Screening of ten cytochrome P450 enzyme activities with 12 probe substrates in human liver microsomes using cocktail incubation and liquid chromatography-tandem mass spectrometry. Biopharm Drug Dispos *40*, 101-111. 10.1002/bdd.2174.

4. Demetriades, M., Leung, I.K., Chowdhury, R., Chan, M.C., McDonough, M.A., Yeoh, K.K., Tian, Y.M., Claridge, T.D., Ratcliffe, P.J., Woon, E.C., and Schofield, C.J. (2012). Dynamic combinatorial chemistry employing boronic acids/boronate esters leads to potent oxygenase inhibitors. Angew Chem Int Ed Engl *51*, 6672-6675. 10.1002/anie.201202000.

5. Trott, O., and Olson, A.J. (2010). AutoDock Vina: improving the speed and accuracy of docking with a new scoring function, efficient optimization, and multithreading. J Comput Chem *31*, 455-461. 10.1002/jcc.21334.

6. Pettersen, E.F., Goddard, T.D., Huang, C.C., Couch, G.S., Greenblatt, D.M., Meng, E.C., and Ferrin, T.E. (2004). UCSF Chimera--a visualization system for exploratory research and analysis. J Comput Chem *25*, 1605-1612. 10.1002/jcc.20084.

7. Wallace, A.C., Laskowski, R.A., and Thornton, J.M. (1995). LIGPLOT: a program to generate schematic diagrams of protein-ligand interactions. Protein Eng *8*, 127-134. 10.1093/protein/8.2.127.

8. Lagorce, D., Douguet, D., Miteva, M.A., and Villoutreix, B.O. (2017). Computational analysis of calculated physicochemical and ADMET properties of protein-protein interaction inhibitors. Scientific Reports *7*, 46277. 10.1038/srep46277.

9. Wagner, G. (1990). NMR investigations of protein structure. Progress in Nuclear Magnetic Resonance Spectroscopy *22*, 101-139. <https://doi.org/10.1016/0079-6565(90)80003-Z>.
